# Remnants of the Legume Ancestral Genome Preserved in Gene-Rich Regions: Insights from *Lupinus angustifolius* Physical, Genetic, and Comparative Mapping

**DOI:** 10.1007/s11105-014-0730-4

**Published:** 2014-05-15

**Authors:** Michał Książkiewicz, Andrzej Zielezinski, Katarzyna Wyrwa, Anna Szczepaniak, Sandra Rychel, Wojciech Karlowski, Bogdan Wolko, Barbara Naganowska

**Affiliations:** 1 Department of Genomics, Institute of Plant Genetics of the Polish Academy of Sciences, Strzeszyńska 34, 60-479 Poznan, Poland; 2 Institute of Molecular Biology and Biotechnology, Adam Mickiewicz University, Umultowska 89, 61-614 Poznan, Poland

**Keywords:** Genome, Synteny, Sequencing, BAC-FISH, Molecular marker, Narrow-leafed lupin

## Abstract

The narrow-leafed lupin (*Lupinus angustifolius*) was recently considered as a legume reference species. Genetic resources have been developed, including a draft genome sequence, linkage maps, nuclear DNA libraries, and cytogenetic chromosome-specific landmarks. Here, we used a complex approach, involving DNA fingerprinting, sequencing, genetic mapping, and molecular cytogenetics, to localize and analyze *L. angustifolius* gene-rich regions (GRRs). A *L. angustifolius* genomic bacterial artificial chromosome (BAC) library was screened with short sequence repeat (SSR)-based probes. Selected BACs were fingerprinted and assembled into contigs. BAC-end sequence (BES) annotation allowed us to choose clones for sequencing, targeting GRRs. Additionally, BESs were aligned to the scaffolds of the genome sequence. The genetic map was supplemented with 35 BES-derived markers, distributed in 14 linkage groups and tagging 37 scaffolds. The identified GRRs had an average gene density of 19.6 genes/100 kb and physical-to-genetic distance ratios of 11 to 109 kb/cM. Physical and genetic mapping was supported by multi-BAC-fluorescence in situ hybridization (FISH), and five new linkage groups were assigned to the chromosomes. Syntenic links to the genome sequences of five legume species (*Medicago truncatula*, *Glycine max*, *Lotus japonicus*, *Phaseolus vulgaris*, and *Cajanus cajan*) were identified. The comparative mapping of the two largest lupin GRRs provides novel evidence for ancient duplications in all of the studied species. These regions are conserved among representatives of the main clades of Papilionoideae. Furthermore, despite the complex evolution of legumes, some segments of the nuclear genome were not substantially modified and retained their quasi-ancestral structures. Cytogenetic markers anchored in these regions constitute a platform for heterologous mapping of legume genomes.

**Electronic supplementary material:**

The online version of this article (doi:10.1007/s11105-014-0730-4) contains supplementary material, which is available to authorized users.

## Introduction

The legume family (Fabaceae) comprises about 19,500 species from 750 genera, grouped into three subfamilies (Mimosoideae, Caesalpinioideae, and Papilionoideae). This family is the most remarkable for its wide evolutionary diversification and cosmopolitan distribution. The genus *Lupinus* belongs to the Papilionoideae and encompasses ∼275 species (Hughes and Eastwood 2006). Phylogenetic analyses based on nuclear internal transcribed spacer (ITS) and chloroplast (*trn*L-*trn*F, *rbc*L) DNA sequences classified the genus *Lupinus* as a distinct lineage within the tribe Genisteae (subtribe Lupininae) (Aïnouche et al. 2004). *Lupinus* is believed to have diverged from the other legume genera ∼17 to 22.5 million years ago (Mya) (Lavin et al. 2005; Drummond et al. 2012). Analyses of genetic similarity have identified three centers of species diversity: North America, Central America, and Andean South America; Atlantic South America; and the Mediterranean and northern and eastern African regions (Ainouche and Bayer 1999). Lupin species are separated into two major groups: the “Old World” and “New World” groups. The Old World group contains about 12–15 species; of them, three (including the narrow-leafed lupin, *Lupinus angustifolius*) have been domesticated as crops. Lupin species are cultivated worldwide, not just for animal feed and human consumption but also as green manure improving infertile soil, due to their ability to fix nitrogen through their symbiotic relationship with Bradyrhizobia. They are popular for their high seed protein contents, low alkaloid profiles, and abilities to adapt to a wide range of environmental conditions.

Considerable progress has been made recently in narrow-leafed lupin genomics, and new genetic resources have been developed. First, a microsatellite-anchored fragment length polymorphism (MFLP) method (Yang et al. 2001) was used to develop a complex linkage map (Boersma et al. 2005), and several sets of molecular markers were linked to particular agronomic traits. These include soft seediness (marker MoLi) (Li et al. 2012), reduced pod shattering (TaLi, LeM1, LeM2) (Boersma et al. 2007b; Li et al. 2010), early flowering (KuHM1) (Boersma et al. 2007a), and resistances to various fungal diseases, including anthracnose (AntjM1, AntjM2) (Yang et al. 2004, 2008; You et al. 2005), phomopsis stem blight (PhtjM1, PhtjM2, Ph258M1, Ph258M2) (Yang et al. 2002), and lupin rust (RustM1, RustM2) (Sweetingham et al. 2005). Moreover, a linkage map with gene-based sequence-tagged site (STS) markers was constructed (Nelson et al. 2006), as was a consensus map containing both MFLP and STS markers (Nelson et al. 2010). The development of next-generation sequencing (NGS) technologies has facilitated the low-cost production of large volumes of genomic and transcriptomic sequences. Transcriptome data have been released for two lupin species, *Lupinus albus* and *Lupinus luteus*. The first white lupin gene index (LAGI 1.0) contained 125,821 unique sequences with an average length of 1,155 bp (O’Rourke et al. 2013), while the yellow lupin transcriptome survey yielded an assembly of 55,309 isotigs and 8,741 full-length proteins (Parra-González et al. 2012). NGS was also applied to *L. angustifolius*, where researchers developed new sets of STS markers linked to selected hypothetical genes, such as those believed to confer anthracnose resistance (Yang et al. 2012) and Phomopsis stem blight resistance (Yang et al. 2013a). A draft assembly of the lupin genome was obtained from a whole-genome shotgun sequencing approach, offering 26.9× coverage (Yang et al. 2013b). More genes have been identified in lupin than in other legume species (e.g., *L. japonicus*, *Medicago truncatula*, *Glycine max*, and *Cajanus cajan*), perhaps reflecting additional round(s) of whole-genome duplication in the lineage leading to *Lupinus* as evidenced from previous studies on chromosome number, transcriptome analysis, and preliminary genome annotation (Naganowska et al. 2003; Parra-González et al. 2012; O’Rourke et al. 2013; Yang et al. 2013b).

The opportunities for physical genome mapping, positional gene cloning, and sequencing have been significantly improved by the development of nuclear genome bacterial artificial chromosome (BAC) libraries for two *L. angustifolius* cultivars: Polish cv. Sonet (Kasprzak et al. 2006) and Australian cv. Tanjil (Gao et al. 2011). The cv. Sonet BAC library contains 55,296 clones with an average insert size of 100 kb, representing approximately six haploid genome equivalents, while the cv. Tanjil BAC library contains 111,360 BACs with a similar average insert length (12× genome coverage). BAC-based molecular studies may be facilitated by cytogenetic analysis (i.e., fluorescent in situ hybridization with BAC clones as probes; BAC-FISH), which allows DNA sequences to be directly mapped to chromosomes. BAC-FISH has been largely exploited for locating genomic sequences in plants with small genomes partitioned into tiny, similar chromosomes (Pedrosa et al. 2002; Fonsêca et al. 2010; Findley et al. 2010). Following the construction of the first *L. angustifolius* BAC library (Kasprzak et al. 2006), BAC-FISH was used to perform cytogenetic mapping of the narrow-leafed lupin genome; this study focused on associating linkage groups with the corresponding chromosomes, with the goal of integrating the genetic and cytogenetic maps of *L. angustifolius* (Kaczmarek et al. 2009; Lesniewska et al. 2011). BAC-FISH has also been used to validate and verify BAC-based DNA fingerprinting (Książkiewicz et al. 2013).

As mentioned, many of the available *L. angustifolius* markers were obtained by DNA fingerprinting approaches based on MFLPs (Yang et al. 2001). These sequences contain short sequence repeat (SSR) motifs, predominantly TTG, GTT, and GA. A comprehensive analysis of SSR distribution in the genome of the model legume, *M. truncatula*, showed that the majority of SSRs are located in the non-transcribed fractions of gene-rich regions (GRRs) or within the untranslated portions of transcripts (Mun et al. 2006). The first attempts to screen the narrow-leafed lupin BAC library with MFLP-derived markers yielded numerous positive hybridization signals. However, cytological localization studies revealed that the isolated BAC clones localized to different chromosomes, indicating that such probes are not useful for positional cloning of particular genes (Lesniewska et al. 2011; Książkiewicz et al. 2013). In contrast, probes based on MFLP-derived markers have been shown to serve as anchor points for tagging of GRRs containing particular SSR motifs; such markers have been proven useful for identifying GRRs in the narrow-leafed lupin genome and have aided in general genomic and syntenic studies of the species (Książkiewicz et al. 2013).

Here, we selected narrow-leafed lupin GRRs from a BAC library by hybridization with four MFLP-derived markers and used diverse molecular methods (e.g., DNA fingerprinting, BAC-FISH, and genetic mapping) to characterize the structure and organization of these regions of the *L. angustifolius* genome. Furthermore, we comprehensively annotated the sequences of selected GRRs and confirmed the results by comparative mapping to gene indexes of *L. albus* and *L. luteus* and expressed sequence tag (EST) databases of Fabaceae, *Glycine* spp., *Lotus* spp., *Medicago* spp., and *Phaseolus* spp. Finally, we identified syntenic and homologous links between *L. angustifolius* and five sequenced legume species representing diverse clades: *M. truncatula*, *G. max*, *Lotus japonicus*, *Phaseolus vulgaris*, and *C. cajan*.

## Materials and Methods

### Hybridization Probes and BAC Library Screening

The hybridization probes were based on the sequences of the MFLP-derived genetic markers, AntjM1, AntjM2 (Yang et al. 2004; You et al. 2005), Ph258M2 (Yang et al. 2002), and RustM1 (Sweetingham et al. 2005) (Hua’an Yang, unpublished). The PCR primers for probe amplification were designed to match the appropriate SSR motifs. The probe sequences were tested for the presence of repetitive elements (BLASTN) and protein-coding regions (BLASTX), with the *e* value cutoffs set to 10^−11^. The BLASTN algorithm was optimized for somewhat similar sequences (word size, 11; match/mismatch scores, 2/−3; and gap existence/extension costs, 5/2). The following parameters were applied to the BLASTX algorithm: word size, 3; matrix, BLOSUM 62; and gap existence/extension costs, 11/1. All probes were PCR amplified using *L. angustifolius* genomic DNA as the template. The resulting PCR products were purified (QIAquick PCR Purification Kit; Qiagen), sequenced to confirm locus-specific amplification (ABI PRISM 3130 XL Genetic Analyzer; Applied Biosystems, Hitachi), and radiolabeled by random priming (HexaLabel DNA Labeling Kit; Fermentas) in the presence of 50 μCi [α-32P]-dCTP. The probe sizes, primer sequences, and SSR loci identified in the probe sequences are given in Table 1. High-density DNA macroarrays containing clones from the *L. angustifolius* nuclear genome BAC library were prepared (GeneTAC G3; Genomics Solutions) on Hybond N^+^ 22.2 × 22.2-cm nylon filters (AP Biotech, Little Chalfont, UK). Probe hybridization, clone selection, and DNA isolation were carried out as previously described (Książkiewicz et al. 2013).

**Table 1:** The sizes and sequences of the library screening probes, PCR primers, and SSR loci identified in the probe sequences

Probe	SSR locus	PCR primers	Probe size
AntjM1	(TTG)_6_	CCCATTGTTGTTGTTG CATCCTCACATATGAAGC	276
AntjM2	(GA)_2_(N)_n_ (GA)_2_(N)_n_ (GA)_2_	GTATCTGATGACAATTAGTCAC TCATCTCTAAATCCTATCTCAG	429
Ph258M2	(GTT)_6_	GGGAACAACAACAACAACAAC GTAGTGACTGAAGAAACTTACAC	240
RustM1	(TTC)_3_ (TTG)_3_	TAACATTCCTACCTTCTT AACACTAGTGCTTCAAAAA	280

### Sequencing of BAC Ends

A PhasePrep BAC DNA Kit (Sigma) was used to isolate bacterial DNA, and the BAC ends were sequenced using the following pIndigoBAC5 (Epicentre, Illumina) sequencing primers: 5′ end, CTCGTATGTTGTGTGGAATTGTGAGC, and 3′ end, GGATGTGCTGCAAGGCGATTAAGTTGG. Chromas Lite 2.01 (Technelysium Pty Ltd) was used to verify the chromatograms and identify mis-call sequencing errors. The BAC-end sequences (BESs) obtained using the 3′ and 5′ primers were given the “_3” and “_5” suffixes, respectively.

#### Restriction Fingerprinting and Contig Assembly

Two units of *Eco*130I and *Hin*dIII were separately used to digest 1 μg of BAC DNA at 37 °C for 16 h. The digestion products were separated by 1 % agarose gel electrophoresis (24 h, 3 V/cm, 8 °C) and visualized by ethidium bromide staining. Normalized band position files were generated using the Image 3.10b gel processing program (Sulston et al. 1989). Products derived from the vector DNA were removed, and BAC contigs were assembled using FingerPrinted Contigs version 8.5.3 (Soderlund et al. 1997), with the following parameters: cutoff 1e-11 and tolerance 3. Additionally, Sequencher 4.7 (Gene Codes) was used to align BESs to tag clones that overlapped at their ends. Furthermore, BESs were used to screen the *L. angustifolius* whole-genome shotgun contig collection deposited in NCBI sequence database (Project No. PRJNA179231; assembly version GCA_000338175.1; subsequent sequence accessions, AOCW01000001 to AOCW01191454). A sequence identity cutoff value of 99 % was applied, and the BLAST algorithm was optimized for highly similar sequences (word size, 28; match/mismatch scores, 1/−2; and gap costs, linear). If two or more BESs were localized to a single scaffold, the appropriate BAC clones were considered to physically overlap.

#### Functional Annotation

The functional annotations of the genetic elements encoded in BACs and BESs included de novo detection of specific signals and comparative analyses with known sequences, as applied using a CEL analysis pipeline specifically designed for gene discovery and comparative genome research (Zielezinski et al. 2012). Prior to gene prediction, transposable element-related repeats were annotated and masked using RepeatMasker 4.0.3 (http://www.repeatmasker.org) and the RepBase 17.11 library (Jurka et al. 2005). Custom Python scripts were written to identify simple tandem repeats (1–6 bp in length). We did not mask simple repeats or microsatellites.

In silico gene prediction was performed using Fgenesh (Salamov and Solovyev 2000) and Augustus (Stanke and Morgenstern 2005). Sequences were subjected to sequence homology searches against the transcriptome sequences of yellow lupin (*L. luteus*) young leaves, buds, flowers, and seeds (Parra-González et al. 2012) and white lupin (*L. albus*) roots and leaves (O’Rourke et al. 2013). The following repositories were selected to download the sequence data: *L. luteus*, http://www.cgna.cl/lupinus (project PRJNA170318, sequence read archive SRX159101); *L. albus*, http://comparative-legumes.org (gene index LAGI 1.0). For each BAC/BES, ESTs and cDNAs with 95 % identity were obtained from the raw transcriptome data of *L. albus* and *L. luteus* and from EST collections representing Fabaceae, *Glycine* spp., *Lotus* spp., *Medicago* spp., and *Phaseolus* spp. The EST collections were screened for vector sequences using cross_match from the Phred package (Ewing et al. 1998), and vector and low quality sequences were trimmed using the NCBI UniVec database as a reference (“FTP site for UniVec,” n.d.). The cleaned reads were initially assembled using CAP3 (Huang and Madan 1999), and the EstScan program (Iseli et al. 1999) was used to scan the EST contigs for potential frameshift errors by detecting irregularities in the coding potential. EST/cDNA contigs were mapped to the genome sequence using the Sim4 program (Florea et al. 1998). BLASTX was used to examine similarities with curated plant proteins in the SwissProt and RefSeq databases, as well as predicted proteins in trEMBL. For the potential genes, gene prediction models were visualized, manually verified, and refined using the Apollo Genome Annotation and Curation Tool 1.11.7 (Lewis et al. 2002).

### Microsynteny Analysis

BAC sequences were masked for repetitive contents and low-complexity regions and then subjected to sequence homology searches against the following genome sequences: *M. truncatula* (Young et al. 2011) (strain A17, JCVI v3.5.4 unmasked, http://www.jcvi.org/medicago/), *L. japonicus* (Sato et al. 2008) (v2.5 unmasked, http://www.kazusa.or.jp), *G. max* (Schmutz et al. 2010) (JGI v1.1 unmasked, http://www.phytozome.net), *P. vulgaris* (v0.9, DOE-JGI and USDA-NIFA, http://www.phytozome.net), and *C. cajan* (Varshney et al. 2012) (project PRJNA72815, v1.0). Sequence similarity analyses were performed using the CoGe BLAST algorithm (Lyons et al. 2008) with the following parameters: *e* value cutoff, 1e-20; word size, 8; gap existence cost, 5; gap elongation cost, 2; nucleotide match score, 1; and nucleotide mismatch score, −2. Syntenic blocks were visualized using the Web-based Genome Synteny Viewer (Revanna et al. 2011) and Circos (Krzywinski et al. 2009).

### Genetic Mapping

Annotated BES and BAC sequences were used to design PCR primers for amplification of DNA isolated from the parental lines of the *L. angustifolius* mapping population: 83A:476 (D) and P27255 (W). When primers yielded single products, amplicons were recovered directly from the post-reaction mixtures (QIAquick PCR Purification Kit; Qiagen). When two or more PCR products were obtained from a primer pair, the relevant DNA bands were excised from the gel and extracted (QIAquick Gel Extraction Kit; Qiagen). Purified amplicons were sequenced. Length polymorphisms were visualized by 1 % agarose gel electrophoresis, and nucleotide substitution polymorphisms were detected by the Cleaved Amplified Polymorphic Sequence (CAPS) or derived CAPS (dCAPS) approaches. Restriction sites were identified using dCAPS Finder 2.0 (Neff et al. 2002). Restriction products were separated by 1–3 % agarose gel electrophoresis, with the agarose concentration adjusted according to the size of the expected digestion products. The mapping population consisted of 90 recombinant inbred lines (F_8_) (kindly provided by Dr. Hua’an Yang, Department of Agriculture and Food, Western Australia). The new markers were localized on the *L. angustifolius* genetic map (Nelson et al. 2010) along with the previously reported markers (Książkiewicz et al. 2013). Linkage mapping was done in Map Manager QTXb20 (Manly et al. 2001). Graphic illustration of linkage groups was performed using MapChart (Voorrips 2002).

### PCR Conditions

The primers were designed using Primer3Plus (Untergasser et al. 2007). Each PCR reaction was performed in a total volume of 20 μl in 96-well twin.tec PCR plates (Eppendorf) using 0.5 U Taq DNA Polymerase Recombinant (Invitrogen), 1× PCR buffer, 2 mM Mg^2+^, 0.25 mM dNTP, 0.25 μM each primer, 50 ng DNA template, and deionized water. The amplification protocol included an initial denaturation at 94 °C for 4 min, followed by 35 cycles of annealing (45–62 °C for 30 s), elongation (72 °C for 40 s) and denaturation (94 °C for 30 s), and a final elongation step (72 °C for 6 min).

### BAC-FISH

DNA was isolated from single *Escherichia coli* colonies (QIAprep Spin Miniprep Kit; Qiagen) (Farrar and Donnison 2007), labeled with digoxygenin-11-dUTP and/or tetramethylrhodamine-5-dUTP (Roche Diagnostics) by nick translation, and used as molecular probes for BAC-FISH. In some cases, two or three BAC clones were simultaneously analyzed in various combinations (multi-BAC-FISH). These studies were carried out on mitotic metaphase chromosomes. Cytological preparations were made from root meristematic tissues, as previously described (Lesniewska et al. 2011). Slide quality was controlled by observation under a phase-contrast microscope (BX41; Olympus). FISH was performed according to the protocol previously adapted for use in *L. angustifolius* (Lesniewska et al. 2011; Książkiewicz et al. 2013). Digoxygenated DNA probes were detected with FITC-conjugated antidigoxigenin primary antibodies (Roche Diagnostics). Chromosomes were counterstained with 2 μg/ml 4',6-diamidino-2-phenylindole (DAPI) (Sigma) in Vectashield antifade mounting medium (Vector Laboratories, Burlingame, CA). Preparations were examined under a BX 60 microscope (Olympus) using the Cell_F software (Olympus). The images were captured using a CCD monochromatic camera and superimposed using Micrografx Picture Publisher 8 software (Corel).

## Results and Discussion

### BAC Library Screening

Hybridization probes were developed from the sequences of four MFLP-derived markers containing particular SSR motifs (AntjM1, AntjM2, Ph258M2, and RustM1) (Yang et al. 2002, 2004; Sweetingham et al. 2005; You et al. 2005) (Hua’an Yang, unpublished) and used to screen the genomic BAC library of *L. angustifolius* cv. Sonet (Table 1). BLAST was used to test the probe sequences for the presence of repetitive elements and protein-coding regions. Several alignments to *M. truncatula* genomic sequences were identified in AntjM1 and RustM1, while Ph258M2 was found to contain a sequence similar to the *Pisum sativum* CONSTANS-like gene (Table 2). The hybridization yielded numerous positive signals, and a total of 124 BAC clones were selected. The vast majority of clones displayed hybridization signals jointly with two or more probes, whereas relatively few hybridized exclusively to one probe (Table 3). The underlying cause of this substantial cross-hybridization could be the presence of analogous repeats in probe sequences, like (TTG)_6_ in AntjM1 and (GTT)_6_ in Ph258M2. However, it does not explain the phenomenon of cross-hybridization to AnjtM2 marker, carrying different repeats, namely (GA)n.

**Table 2:** Nucleotide and protein accessions identified in the designed hybridization probes

Probe	Identified sequence	*E* value
AntjM1	AC225502.9, *M. truncatula* clone mth2-58c24	2e-22
Ph258M2	AY830921.1, *Pisum sativum* CONSTANS-like	1e-37
RustM1	AC147007.17, *M. truncatula* clone mth2-26c3	2e-53

**Table 3:** The number of positive signals obtained after probe hybridization of the BAC library

	AntjM1	AntjM2	Ph258M2	RustM1
Total signals	64	115	108	105
Unique signals	0	1	1	3
Joint signals	60^a^		94^b^	
Joint signals	46^c^			

^a^a number of signals shared by AntjM1 and AntjM2 probes

^b^a number of signals shared by Ph258M2 and RustM1 probes

^c^a number of positive signals joint for all applied probes

All of the selected BAC clones were end-sequenced. Sequencing from the 5′ end failed for clones 024B21, 024F17, 042B18, and 114F10, whereas that from the 3′ end failed for clone 140L16. We obtained 243 BESs with an average insert length of 665 bp; they have been deposited as Genome Survey Sequences in the DNA Data Bank of Japan (DDBJ; accessions AB809166 to AB809361) and in GenBank at the National Center for Biotechnology Information (NCBI; accessions HR864171 to HR864217).

### Physical Mapping of BAC Clones

To examine their putative clustering in the lupin genome, the BAC clones were subjected to restriction enzyme DNA fingerprinting. Initially, DNA isolated from five BAC clones was digested separately with *Vsp*I, *Bsh*I, *Eco*130I, *Acy*I, *Bam*HI, *Xba*I, *Xho*I, and *Hin*dIII and double-digested with *Xba*I and *Xho*I. From this, we selected two enzymes that each generated 20–40 products (*Eco*130I and *Hin*dIII) and used them to fingerprint the entire set of BAC clones. The FingerPrinted Contigs (FPC) assembly procedure utilized in the present work depends mainly on two parameters: tolerance and cutoff. These variables and their adjustment methods were described in a previous methodology paper (Soderlund et al. 1997); here, we used a tolerance of 3 and a cutoff of 10^–11^. Analogous agarose gel-based approaches for contig construction used values ranging from 3 to 7 for tolerance and 10^−8^ to 10^−16^ for cutoff (Marra et al. 1997; Chen et al. 2002; Ng et al. 2005). Based on the restriction patterns, we constructed 14 contigs containing a total of 97 clones, whereas 27 clones failed to group and were designated as singletons. Contig 1, which was visualized by 9 “Q” clones with unclear contig positions, was found to suffer from instability. A disproportionate number of Q clones might reflect improper assembly of repetitive sequences (Katagiri et al. 2005). Calculations based on restriction product sizes and previous pulsed field gel electrophoresis (PFGE) results (Lesniewska et al. 2011) enabled us to estimate the physical length of FPC “consensus band” as 3.0 kb.

Furthermore, to identify BAC clones that overlapped directly at their ends, comparative analysis of BESs was performed. This approach entirely confirmed the grouping of 32 clones into 10 contigs. Next, we used BLASTN to align all of the BESs to *L. angustifolius* whole-genome shotgun contigs and identified corresponding scaffolds for 121 BESs. These assignments were very specific under the utilized cutoff and BLAST algorithm parameters. No BES localized to more than one scaffold, while six scaffolds harbored BESs originating from two or more clones. Furthermore, our analysis showed that 11 BACs physically overlapped in five contigs (data not shown).

### Functional Annotation of BAC-End Sequences

The generated BES collection (161,697 bp in total) was subjected to in silico annotation of various genetic elements. Our initial analysis identified 38.3 % of the BESs as repetitive sequences; of them, 58.7 % were retrotransposons and 11.4 % were transposons. The first group of transposable elements (TEs) included predominantly Ty1/Copia (63.1 %) and Ty3/Gypsy (25.1 %), whereas the second subset (the transposon group) was represented mainly by hAT (77.9 %) and CMC-EnSpm (20.2 %). In addition to the TEs, the BES collection contained a “repetitive” subset that consisted of simple repeats (26.9 %) and rRNA (3.11 %). Online Resource 1 contains data on the RepeatMasker and Repbase annotations, along with the relevant sequence coordinates and alignment scores. During the second step of annotation, we identified several sequences with similarities to known genes encoding peroxidases, Nudix hydrolase, senescence-associated proteins, cytochrome b5 reductase, tubulins, callose synthase, tetraspanin, pathogenesis-related proteins, multidrug resistance proteins, lectin, and leghemoglobin. In general, 27.3 % of total BES content was annotated as gene-coding.

**Fig. 1:**
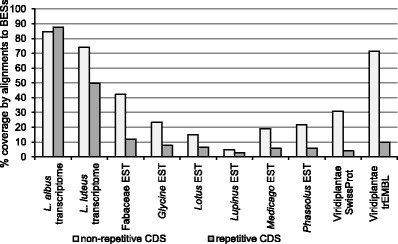
Percentage BES coverage of EST, SwissProt, and trEMBL sequence alignments. Coding DNA Sequences (CDSs) identified in BESs were annotated using repeat element collections and classified as repetitive or non-repetitive. The BES-derived CDSs were then aligned to the indicated sequence databases, and the percent coverage was calculated

The BESs were also aligned to recently published transcriptome data from *L. albus* and *L. luteus*; the ESTs of Fabaceae, *Glycine*, *Lotus*, *Lupinus*, *Medicago*, and *Phaseolus*; and the Viridiplantae SwissProt and trEMBL databases (Fig. 1). In the fraction of BESs assigned to the TE subgroup, the highest alignment coverages were observed for the transcriptomes of *L. albus* (84.6 %) and *L. luteus* (74.0 %) and for Viridiplantae trEMBL (71.3 %). Sequences from Fabaceae dbEST GenBank database produced alignments for 42.2 % of the TE-assigned BESs, with the highest coverages noted for the ESTs of *Glycine* (23.3 %), *Phaseolus* (21.9 %), and *Medicago* (19.1 %). Among the non-repetitive gene-coding BESs, high alignment coverages were obtained only for the lupin transcriptome data of *L. albus* (87.7 %) and *L. luteus* (49.7 %), while Fabaceae EST hits were identified for 11.9 % of the gene-coding BESs. Detailed BES annotation data, including sequence coordinates, alignment quality *e* values, ID numbers, and accessions, are given in Online Resource 1.

### BAC Clone Sequencing

BAC clones were selected for whole-insert sequencing based on their BES annotations to target-gene-rich (four clones) and repetitive (one clone) regions of genome and included clones 017B07 (5′ end, callose synthase; 3′ end, ABC transporter), 075D16 (5′ end, Nudix hydrolase; 3′ end, heme oxygenase), 112N18 (5′ end, ABC transporter; 3′ end, ethanolamine kinase), 119M23 (5′ end, transposon CMC-EnSpm; 3′ end, retrotransposon Ty1/Copia), and 136B16 (5′ end, very-long-chain fatty acid condensing enzyme; 3′ end, alpha-tubulin). Next-generation sequencing (454) allowed us to construct three contigs for 017B07 (109,008 bp), eight for 075D16 (98,086 bp), three each for 112N18 and 119M23 (43,842 and 97,240 bp), and four for 136B16 (103,792 bp). The contigs corresponding to BAC clones 017B17, 112N18, and 119M23 were ordered and oriented according to the results of our BES alignments and PCR amplification with contig-derived primers. For clones 075D16 and 136B16, only BES-containing external contigs were ordered and oriented.

### BAC Clone Annotation

Annotation revealed that the frequencies of interspersed repeats (not including SSRs) in the sequenced BACs varied from 1.1 % in clone 017B07 to 37.7 % in clone 119M23 (Table 4). We observed a high prevalence of retrotransposons, particularly Ty1/Copia. Transposon occupancy was negligible, and the occupied sequences consisted of just two families, hAT and CMC-EnSpm.

**Table 4:** Summary of the BES, BAC, and scaffold sequence annotations

Sequence	BES	017B07	075D16	112N18	119M23	136B16	Scaffold	Total
DNA/EnSpm	0.88^a^	–	–	–	0.82	–	0.09	0.18
DNA/hAT	3.39	–	1.95	–	–	0.64	0.04	0.46
DNA/Helitron	0.08	–	–	–	–	–	0.19	0.13
DNA/other	–	–	–	–	–	–	0.21	0.14
LTR/Copia	14.15	0.08	22.00	6.90	36.85	14.83	11.73	13.18
LTR/Gypsy	5.62	0.35	1.73	10.94	–	7.94	7.15	6.09
LTR/other	0.03	–	–	–	–	–	0.22	0.15
Non-LTR/SINE	–	0.11	–	–	–	–	–	0.01
Non-LTR/LINE	1.93	–	–	–	–	–	1.28	1.02
Non-LTR/RTE	0.70	0.56	0.07	–	–	0.64	0.39	0.39
rRNA	1.19	–	–	–	–	–	0.39	0.36
Simple repeat	10.28	13.80	12.21	15.51	12.13	13.24	9.72	10.60
Total repeats	38.25	14.90	37.96	33.35	49.80	37.29	31.41	32.71
Total genes EST-confirmed non-repetitive	27.30	64.39	2.67	14.16	–	16.81	18.66	20.07

– no alignment was found, *DNA* class II TEs (transposons), *LTR* class I TEs (retrotransposons) with long terminal repeats, *non-LTR* class I TEs (retrotransposons) lacking long terminal repeats

^a^Percentage of sequence covered by alignments to particular elements

**Fig. 2:**
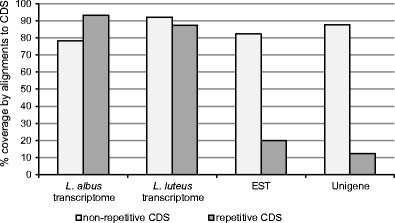
Percentage BAC coverage of EST and Unigene sequence alignments. CDSs identified in BAC sequences were annotated using repeat element collections and classified as repetitive or non-repetitive. The BAC-derived CDSs were then aligned to the indicated sequence databases, and the percent coverage was calculated

In the five sequenced BAC clones, we identified a total of 30 genes that were not related to repetitive elements. A predominance of non-repetitive, EST-confirmed genes was observed in clone 017B07 (64.4 %). This clone represents a GRR with an estimated gene density of 19.3 genes/100 kb. Clones 112N18 and 136B16 showed moderate coverage by gene-coding sequences (14.2 and 16.8 %, respectively). Our analysis of lupin transcriptome data identified statistically significant alignments for all 30 genes in *L. albus* and for 28 genes in *L. luteus*. EST-NCBI and Unigene representatives were identified for all of the annotated genes. The constructed alignments covered 90.1 % of the hypothetical gene transcript lengths on average (95.4 % in *L. luteus*, 88.4 % in *L. albus*, 85.9 % in EST-NCBI, and 90.8 % in Unigene) (Fig. 2). Comparison of annotated gene sequences to selected reference accessions allowed us to perform in silico translation of the complete protein sequences for 14 genes and partial protein sequences (>50 % of the reference sequence length) for another eight genes. Detailed data on the BAC annotation, including predicted genes, ESTs (*L. albus*, *L. luteus*, NCBI in general), Unigene coverage, protein coverage, reference accessions, gene coordinates, exon coordinates, and sequences are shown in Online Resource 2. The BAC clone sequences and their annotation data have been stored in the High Throughput Genomic Sequences Division of the European Molecular Biology Laboratory under project PRJEB1600 (accessions HF937076 to HF937080).

### Reference to the Draft Lupin Genome Assembly

BESs were aligned to the scaffolds and contigs of the narrow-leafed lupin genome draft sequence (Yang et al. 2013b), where they tagged 114 sequences in total (Online Resource 3). The orientations of 74 scaffolds were identified by paired BESs. The lengths of the scaffolds varied from 646 to 74,051 bp. The sequences of 55 scaffolds longer than 10 kb (1,241,568 bp in total) were functionally annotated to supplement the information obtained from our analysis of BESs and BACs. On average, 21.7 % of the scaffold sequences were annotated as TEs; however, this value varied considerably by scaffold (from 0 to 53.7 %). As observed in the BESs and BACs, retro elements were the most abundant, represented mainly by the Copia and Gypsy subclasses, followed by LINEs and RTEs (Table 4). The transposon class was mainly represented by the Helitron, CMC-EnSpm, and (rarely) hAT groups. The functional annotation of BAC-end sequences and adjacent scaffolds of the contig 1 revealed a large subset of transposon and retrotransposon elements. The presence of such repetitive sequences may explain the incorrect assembly of pseudo-contig 1.

BAC and scaffold sequences with unmasked repetitive elements were aligned to the genome sequences of five legume species (*M. truncatula*, *G. max*, *L. japonicus*, *P. vulgaris*, and *C. cajan*) to determine the expansion profiles of such sequences in the Papilionoid clades. Several types of distribution patterns were identified, showing species-specific differences in the presence and abundance of particular repeats (Online Resource 4). The most common pattern, obtained for 35 % of Copia and 38 % of Gypsy elements, was a fairly ubiquitous distribution of alignments across numerous chromosomes in all tested species. The second most frequent pattern, which was observed for 20 % of Copia elements (but no Gypsy element), was the presence of numerous alignments in the genome of *G. max*, with few or no copies observed in the other species. In the remaining cases, we observed numerous alignments to two to four species; this was frequently seen for Copia and Gypsy elements, as well as for less abundant repeats, such as hAT, Harbinger, RTE1, and LINE repeats. Assessment of the repeat sequence distribution within legume genomes revealed that 99 % of the analyzed lupin repeats yielded alignments in the *G. max* genome, 76 % in *C. cajan*, 68 % in *P. vulgaris*, 57 % in *L. japonicus*, and 51 % in *M. truncatula*. These differences indicate that the sequence-level conservation of interspersed repeats is somewhat higher between *Lupinus* and the Phaseoleae than between *Lupinus* and representative Loteae or Trifoliae.

The in silico detection of coding regions in scaffolds revealed a total of 289 genes. Comparisons to repeat sequences in various databases allowed use to identify 153 non-repetitive genes among them. The average gene density was 8.2 genes/100 kb of sequence. As many as 18 scaffolds were classified as GRRs, with gene densities >15 genes/100 kb (range from 15.5 to 31.8 genes/100 kb). The annotation data for the scaffolds are presented in Online Resource 5.

### Simple Repeats

All sequences generated in the study and the scaffolds carrying the selected BESs (1,855,233 bp in total) were analyzed for the presence of SSRs. Repeats differing by reading frames (e.g., AG vs. GA) or reverse-complement reading were clustered. The most frequent were mononucleotide tracts, in which A/T was 10-fold more common than C/G. Approximately half of the dinucleotide repeats were AT, while more than half of the trinucleotide repeats were AAT. The other major clusters of di- and trinucleotide repeats corresponded to AG, AAC, and AAG; these included the SSRs harbored within the designed probe sequences (TTG, TTC, GA). The analysis of BAC sequences and paired-end scaffolds revealed that some BACs contained SSRs of two or more probe sequences. It could be the reason of cross-hybridization between probes carrying non-analogous repeats. Tetranucleotides were represented mainly by repeats containing one C/G nucleotide and three A/T nucleotides (Table 5). The frequency of SSRs in the analyzed sequences was much higher than that previously observed among randomly selected BESs (Gao et al. 2011). This indicates that the use of SSR-anchored probes allowed us to effectively target regions of the lupin genome that contained such sequences. In general, SSRs (except for mononucleotides and AT dinucleotides) are located in GRRs (Mun et al. 2006). In rice, dinucleotide repeats of (GA)_n_ usually occur in gene-flanking regions and do not appear to be commonly associated with transposable elements (Temnykh et al. 2001). Mining of *Brassica rapa* ESTs revealed that (GA)_n_ and (AG)_n_ repeats comprise 13.3 % of all EST-SSRs (Ramchiary et al. 2011), whereas the majority of trinucleotide repeats in the gene-coding regions of the *Arabidopsis* genome were found to be AT-rich (Cardle et al. 2000). The in silico mapping of (GTT)_n_ microsatellites in the soybean identified 32 sites located in high-gene-density regions but only one site in a gene-free region (Belarmino et al. 2012). Our results are consistent with those of the earlier studies, as the majority of SSR loci identified in this survey were localized in regions with gene densities higher than 10 genes/100 kb.

**Table 5:** Percentage of simple repeats identified in the analyzed sequences

Type	Percentage	Type	Percentage
Mononucleotide	**94.93** ^**a**^	Tetranucleotide	**0.13**
A/T	91.44^b^	AATG	29.63
C/G	8.56	AAAG	16.67
Dinucleotide	**2.45**	ATAC	14.81
AT	49.55	AACT	14.81
AG	30.60	AATA	12.96
AC	19.85	AATT	11.11
Trinucleotide	**2.38**	Other	19.40
AAT	55.96	Pentanucleotide	**0.01**
AGG	12.04	Hexanucleotide	**0.10**
AAC	11.92	AGGATG	50.58
AAG	11.88	AGGAAG	30.65
ATG	6.28	Other	12.67
Other	1.92		

Bold font indicates percent frequencies of main groups of repeats

^a^Percent frequency of SSR type

^b^Percent frequency of SSR subgroup within the particular SSR type

### BAC-FISH

Fluorescent in situ hybridization was used to support our physical and genetic mapping of the BAC clones. Initially, we used the results of our contig assembly and BES annotation to select clones for cytogenetic analysis. The initial contig-representing BACs yielded repetitive BAC-FISH signals dispersed over numerous chromosomes, so we further analyzed other overlapping clones. We subjected a total of 83 BACs (including 30 singletons) to cytogenetic analysis. By comparison to our BES annotation, these BACs could be categorized as having (a) EST sequences at both ends; (b) TEs at both ends; (c) no significant similarity to ESTs or TEs at either end; (d) ESTs at one end and no similarity at the opposite end; (e) TEs at one end and no similarity at the other end; and (f) TEs at one end and ESTs at the other end. We tested all clones of contigs 1, 3, 4, 6, 7, and 10–13 and selected clones of the remaining contigs (one in contig 14, two in contigs 2 and 8, and three in contig 9). We did not test any BAC from contig 5 because these clones had been comprehensively analyzed in a prior study aimed at assigning the first genetic linkage groups (LGs) to chromosomal maps of *L. angustifolius* (Lesniewska et al. 2011). In the present work, 11 clones produced distinct single-locus signals: one clone from contig 14, two from contigs 2 and 9, four from contig 1, and two singleton BACs. When we combined our results with those previously obtained for contig 5 (four clones with single-locus signals and three with repetitive dispersed signals) (Lesniewska et al. 2011), we found that approximately 17 % of the SSR probe-selected BACs produced single-locus signals in BAC-FISH. The highest percentages of BAC-FISH single-locus clones were observed in sets “f” (26 %), “c” (25 %), and “a” (21 %). Four clones contained rRNA repeats (18S rRNA) at one of their ends; these clones produced single-locus BAC-FISH signals regardless the type of sequence at the opposite end. Thus, we conclude that the presence of a TE or EST sequence at one end of a BAC does not imply that the clone will yield a repetitive or single-locus signal in BAC-FISH. However, all clones that contained TEs at both ends produced repetitive and dispersed signals in our cytogenetic analysis. Thus, when selecting clones for BAC-FISH, BES annotation might be used as an auxiliary step to help sift out clones that are likely to yield repetitive hybridization signals. Of the five clones sequenced, one (017B07) hybridized to a single locus in BAC-FISH, whereas the other four showed repetitive signals. Functional annotation revealed that clones presenting multiple dispersed signals in BAC-FISH carried numerous interspersed repeats that constituted more than 20 % of their insert sequences (Table 4). These results are in line with previous findings (Belarmino et al. 2012; Książkiewicz et al. 2013), illustrating that there is a distinct relationship between the sequence composition of clones (i.e., repetitive content allowance) and their BAC-FISH signal patterns.

Clones that yielded single-locus signals were used for multi-BAC-FISH, a comprehensive cytogenetic analysis in which differently labeled clones are concurrently applied to the same chromosome slide (Table 6). We used mitotic chromosomes, despite the limited axial resolution and high minimal probe size requirements of this technique, because the aim was to localize the clones to individual chromosomes. Furthermore, this strategy allowed us to perform internal contig control. When cytogenetic signals of BACs from the same contig matched at a single chromosomal site, this was taken as supporting the integrity of the contig. This was seen for clones 072O21 and 115G22 from contig 2 and clones 008A03 and 112E21 from contig 9. Conversely, the BAC-FISH localization of clones of a single contig to different chromosomes was taken as negating the physical linkage of these clones, as was demonstrated for clones 015P08, 017B07, 043C18, and 115C21 from contig 1. Moreover, three clones from contig 1 previously hybridized to three different chromosomes (Kaczmarek et al. 2009), further supporting our contention that we observed false-positive overlapping of these clones. Thus, contig 1 does not correctly represent the physical structure of a genomic region. Such a false-positive overlapping was observed only for contig 1.

**Table 6:** Cytogenetic markers of lupin chromosomes: co-localization of clone pairs tested in BAC-FISH

138N02 ctg 0	015P08 ctg 1	017B07 ctg 1	043C18 ctg 1	115C21 ctg 1	072O21^a^ ctg 2	115G22 ctg 2	008A03 ctg 9	112E01 ctg 9	123A20 ctg 14	083C06		
N	–	–	–	–	–	–	–	–	–	–	044J16	NLL-06
	–	–	–	Y	–	–	–	–	N	–	138N02	NLL-14
		N	N	N	–	–	–	–	–	–	015P08	NLL-09
			N	N	N	N	N	N	–	Y	017B07	NLL-20
				–	–	–	–	–	–	–	043C18	NLL-01
					N	N	N	N	–	–	115C21	NLL-14
						Y	Y	Y	–	–	072O21	NLL-16
							Y	Y	–	–	115G22	NLL-16
								Y	–	–	008A03	NLL-16
									–	–	112E01	NLL-16
										–	123A20	-

*ctg* contig localization, *NLL* linkage group assignment, *Y* clones co-localized to the same chromosome, *N* clones localized to different chromosomes

^a^BAC 072O21 was cytogenetically mapped to the *L. angustifolius* chromosomes by K. Lesniewska (unpublished)

### Genetic Mapping

To assign chromosomes to their corresponding linkage groups, clones showing single-locus BAC-FISH signals were used for molecular marker development and genetic mapping. Genetic markers were also generated for clones containing annotated hypothetical genes, with the goal of physically localizing the GRRs in the *L. angustifolius* genome. Seventy-six BESs from 44 BACs were used as templates for primer design (one pair per BES), and the primers were used to amplify DNA isolated from the parental lines of the mapping population: 83A:476 and P27255. PCR products with expected lengths and sequences were obtained for all 72 primer sets. When no polymorphism between parental lines was found at either end of a BAC clone, the BESs were elongated by Sanger sequencing, and subsequent PCR primer sets were prepared. Fourteen BESs underwent one round of extension, six underwent two rounds, and one underwent three rounds. Sequence polymorphisms between parental lines were identified in 43 PCR products. All marker and primer sequences were deposited in the sequence-tagged site databases of the NCBI (accessions GF110936 to GF110969) and DDBJ (accessions AB811081 to AB811182, AB811255 to AB811351, and AB811459 to AB811463).

In total, 35 markers originating from 33 BESs of 28 clones were obtained for use with various detection methods. For nine of the markers, primers anchored in polymorphic loci were designed, and allele-specific PCR (AS-PCR) with a dominant ratio of segregation was performed. Seventeen markers were visualized using the CAPS (Konieczny and Ausubel 1993) approach, as we were able to match the differing nucleotides with the restriction sites of commercially available enzymes. The remaining nine markers were resolved by the dCAPS method, based on the use of mismatch PCR primers to introduce a restriction site into the polymorphic locus. Our scoring of segregation data from the narrow-leafed lupin mapping population and subsequent linkage analysis allowed us to saturate the *L. angustifolius* genetic map (Nelson et al. 2010) with 35 new markers distributed to 14 linkage groups (Online Resource 6). Detailed marker data and segregation scores are given in Online Resource 7.

Genetic mapping of markers anchored in BAC-end sequences allowed us to determine the genetic positions of the vast majority of the non-repetitive genes identified in the BESs and BACs. Furthermore, the developed markers precisely determined the linkage positions of all but one of the contigs and proved useful for verifying the overlap of the BAC clones. BES-based genetic markers were designed for both ends of selected BACs, including the singletons, 131C21 and 060B20, and clones from contigs 3 (clone 015L10), 9 (clone 051C12), and 12 (clone 080K03). Such physically linked markers were co-localized on the narrow-leafed lupin genetic map at distances ranging from 0.6 to 5.9 cM, converging with the accuracy of genetic mapping. Four markers originating from contig 3 (015L10_5D, 111L22_5, 141C03_5D, and 015L10_3) were clustered in linkage group NLL-17, spanning a range of 6 cM. Two markers from contig 8 (136B16_5 and 112N18_3D) were localized in linkage group NLL-14 at a distance of 0.5 cM. Marker 084P14_5, tagging a clone from contig 5, was mapped 1.7 cM away from marker 142D13_3, which was previously developed for the other BAC from this contig (Lesniewska et al. 2011). In contrast, false-positive overlapping of the clones in contig 1 was demonstrated by the localization of genetic markers originating from four BACs in different linkage groups.

The results of genetic mapping came together with the results of BAC-FISH approach presented in this paper as well as by other authors (Kaczmarek et al. 2009; Lesniewska et al. 2011). In the first report aimed at karyotyping the narrow-leafed lupin, clones from contigs 3 (015L10) and 6 (016J01) localized to the same chromosome, whereas a clone from contig 10 (042B18) was placed on another chromosome (Kaczmarek et al. 2009). Furthermore, the results of a prior cytogenetic assignment of narrow-leafed lupin genetic linkage groups to chromosomes indicated that four BAC clones from contig 5 were found in close physical proximity (Lesniewska et al. 2011). Thus, the previous findings are entirely consistent with the physical and genetic mapping results described herein.

### Ratio of Physical to Genetic Distances

The ratio of physical to genetic distances was calculated using genetic linkage distances and consensus band size data. The distances calculated for singletons were as follows: 131C21, 35 kb/cM; 051C12, 109 kb/cM; and 080K03, 95 kb/cM. Those for contigs were as follows: for contig 3, 11 kb/cM; contig 5, 150 kb/cM; contig 8, 210 kb/cM; and contig 12, 167 kb/cM. The physical-to-genetic distance ratio supports the functional annotation procedure, since the recombination frequency is positively correlated with gene density (Chen et al. 2002; Shah and Hassan 2005; Xu et al. 2008). The average physical distance per centimorgan calculated for 245 syntenic loci of *P. vulgaris* and *G. max* was 290 kb; however, 42 % of the comparisons were <100 kb/cM (McClean et al. 2010). Thus, the results from the present and previous studies indicate that 051C12, 080K03, 131C21, contig 3, and contig 5 all originate from GRRs. This conclusion is supported by the functional annotation of lupin genome scaffolds in GenBank. Corresponding scaffolds were identified for several clones from contigs 3 and 5 and for singleton 080K03, which had an average gene density of ∼19.6 genes/100 kb (Online Resource 5). This value is similar to the 17.7 genes/100 kb (31 genes in 175.35 kb) previously found for two GRRs in narrow-leafed lupin (Książkiewicz et al. 2013).

### Integrative Map of the Narrow-Leafed Lupin Genome

**Fig. 3:**
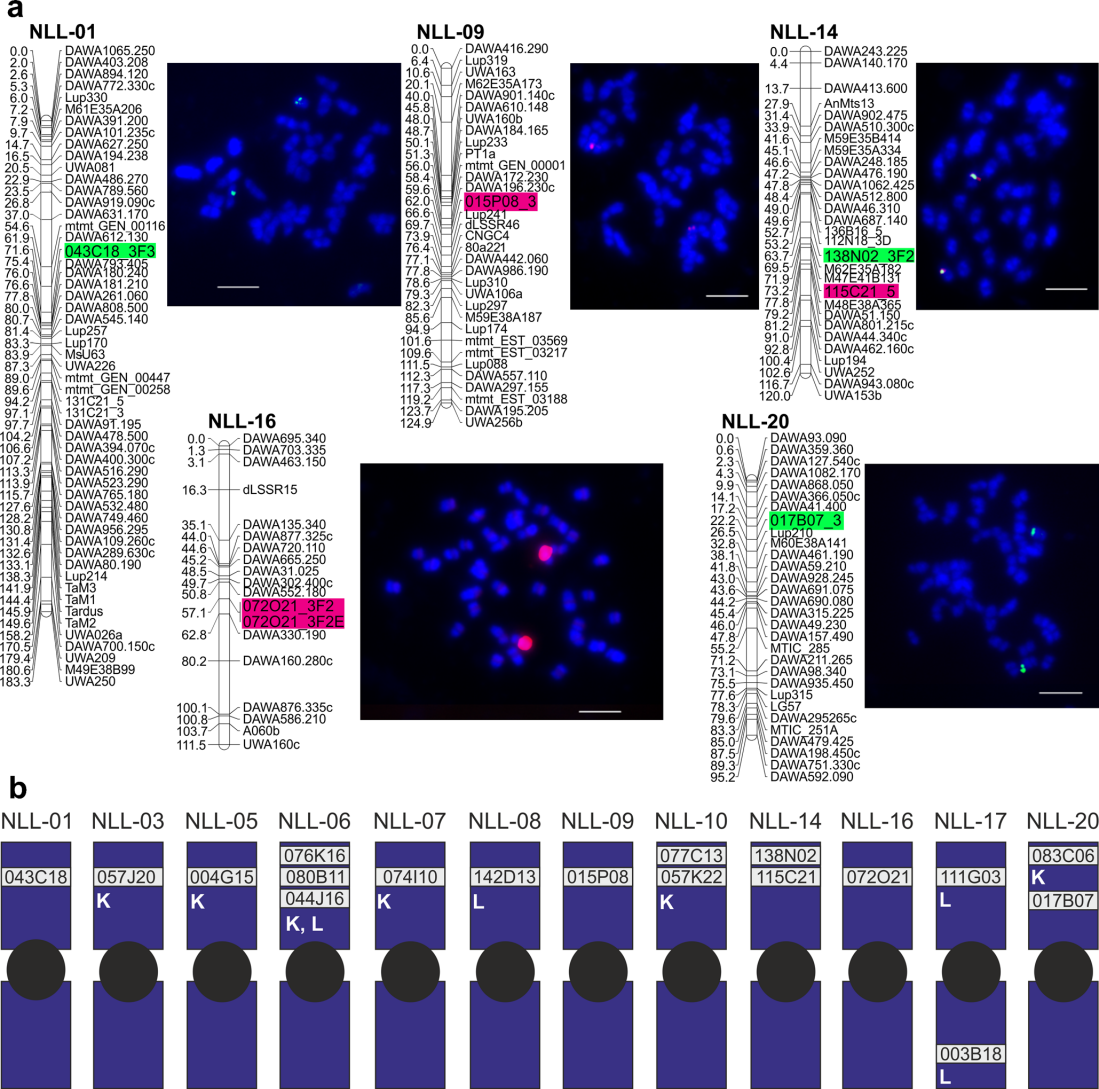
Integrative map of the narrow-leafed lupin genome: BAC-FISH-based assignment of linkage groups to their corresponding chromosomes. **a** Localization of BAC clones 043C18, 015P08, 138N02, 115C21, 072O21, and 017B07 to *L. angustifolius* chromosomes. The genetic positions of the clones are represented by the BES-derived genetic markers, 043C18_3F3, 015P08_3, 138N02_3F3, 115C21_5, 072O21_3F2 + 072O21_3F2E, and 017B07_3, respectively. BAC clone DNA was labeled with tetramethylrhodamine-5-dUTP (*red signals*) or digoxigenin-11-dUPT (*green signals*). Overlapping clones produced yellow signals. Chromosomes were counterstained with DAPI. *Scale bar* = 5 μm. **b** Idiogram of *L. angustifolius* (12 of 20 chromosomes) showing the available BAC chromosome-specific markers, including our novel markers and the previously reported markers (Książkiewicz et al. 2013; Lesniewska et al. 2011)

The use of a complex approach involving diverse nucleic acid based methods (e.g., restriction enzyme DNA fingerprinting, Sanger sequencing, 454 sequencing, genetic mapping, and molecular cytogenetics) enabled us to make considerable progress toward constructing an integrative map of the *L. angustifolius* genome. Five new linkage groups were directly linked to their chromosomes by sequence-specific genetic markers anchored in single-locus BAC clones. Thus, only 7 of 20 chromosome pairs remain unlinked at present (Fig. 3). Additionally, 37 scaffolds were anchored to the reference genetic map of narrow-leafed lupin by BES-derived molecular markers (Online Resource 3). When the draft narrow-leafed lupin genome assembly was released, a total of 4,214 scaffolds were aligned with the genetic map (Yang et al. 2013b); however, the previous study was based on a different mapping population, meaning that our present data cannot be directly transferred. To generate a consensus map that includes linkage data from the germplasm of different parental lines, we need a set of sequence-based anchor markers. The various NGS techniques can produce numerous genetic markers, but they may not be evenly spread throughout the genome. This was observed in the narrow-leafed lupin draft genome assembly, where >50 % of the restriction site-associated DNA sequencing (RAD-seq) markers localized to the four most saturated linkage groups, whereas the six least densely saturated groups collectively accounted for <5 % of markers (Yang et al. 2013b). This uneven distribution of RAD-seq markers across the genome could not be explained by differences in chromosome size because the chromosomes of the narrow-leafed lupin are morphologically uniform, with mean relative lengths fitting in the range of 1.6 to 3.3 % of the genome (Kaczmarek et al. 2009).

### Microsynteny Between the Narrow-Leafed Lupin and Five Reference Legume Species

**Fig. 4:**
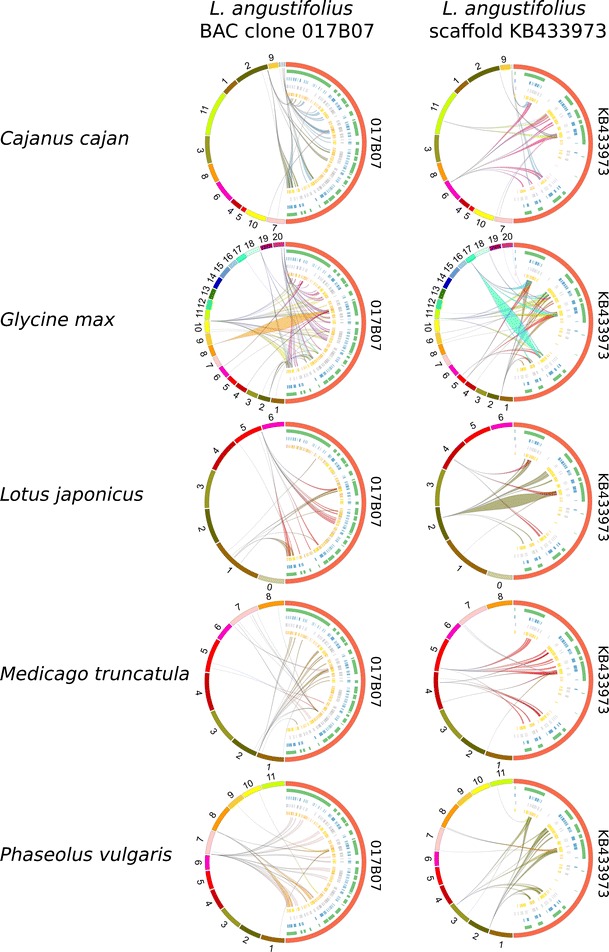
General insight into gene-rich region (GRR) microsynteny between *L. angustifolius* and five model legume species. Two narrow-leafed lupin GRRs are shown: BAC clone 017B07 and scaffold KB433973. Repetitive sequences were masked prior to analysis. Circos (Krzywinski et al. 2009) plots are ordered in rows according to the species (*Cajanus cajan*, *Glycine max*, *Lotus japonicus*, *Medicago truncatula*, and *Phaseolus vulgaris*). Reference legume chromosomes with appropriate numbers are drawn on the *left* of the external ring of each plot, while narrow-leafed lupin regions are shown on the *right*. The annotation data are presented on the internal rings as follows: genes (*green*); Fabaceae GenBank ESTs (*blue*); *L. albus* transcriptomic data (*white*); and *L. luteus* transcriptomic data (*yellow*). Ribbons symbolize homologous links identified by DNA sequence similarity. The chromosomes and corresponding lupin gene-rich regions are not drawn to scale

**Fig. 5:**
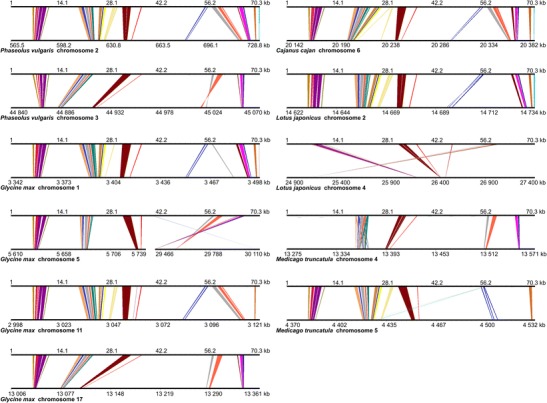
Microsyntenic links between the *L. angustifolius* GRR, KB433973, and sequences from other legumes. Microsyntenic blocks are presented as Genome Synteny Viewer (GSV) (Revanna et al. 2011) diagrams. Each diagram is composed of two horizontal lines; the *upper line* shows the sequence of *L. angustifolius* scaffold KB433973, while the *lower line* shows the corresponding region of a model legume genome. The *scales of the bottom bars* vary by species, so the chromosome localizations are given in kilobases. Homologous links are consecutively colored to portray the order of the syntenic blocks. To simplify the illustration, the following are presented as reverse-complement sequences: *Glycine max* chromosome 1, *Medicago truncatula* chromosome 4, and *Lotus japonicus* chromosomes 2 and 4

**Fig. 6:**
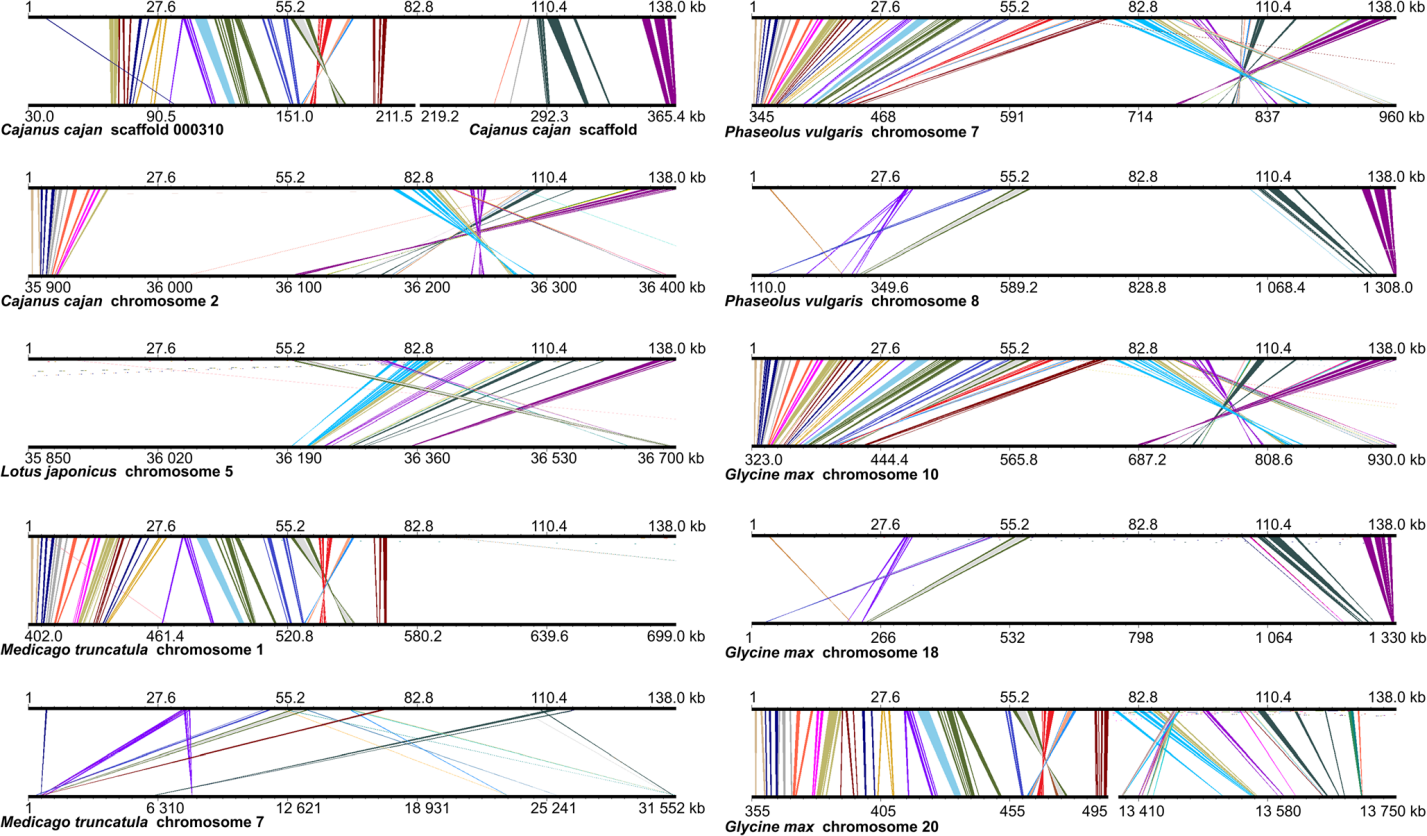
Microsyntenic links between the *L. angustifolius* GRR, 017B07, and sequences from other legumes. Microsyntenic blocks are presented as Genome Synteny Viewer (GSV) (Revanna et al. 2011) diagrams. Each diagram is composed of two horizontal lines; the *upper line* shows the sequence of *L. angustifolius* BAC clone 017B07, while the *lower line* shows the corresponding region of a model legume genome. The *scales of the bottom bars* vary by species, so the chromosome localization coordinates are given in kilobases. Homologous links are consecutively colored to portray the order of the syntenic blocks. To simplify the illustration, the following are presented as reverse-complement sequences: *Cajanus cajan* scaffolds 000310 and 132622; *Glycine max* chromosomes 10, 18, and 20; and *Medicago truncatula* chromosome 1

The BAC and scaffold sequences were subjected to repeat masking and aligned to the genomic sequences of five legume species (*M. truncatula*, *G. max*, *L. japonicus*, *P. vulgaris*, and *C. cajan*), in an effort to evaluate the sequence similarity between species and identify syntenic regions. Syntenic patterns were observed only for the GRRs of clone 017B07 and scaffolds KB415871, KB432947, and KB433973. Scaffold KB432947 was found to overlap with clone 017B07, yielding a 138-kb sequence harboring 24 hypothetical genes. Although we masked the repetitive elements before our analysis, the conserved collinearity between *L. angustifolius* and other legume species was found only in regions characterized by a low occupancy of retrotransposons. Negative correlations between retrotransposon coverage and synteny have been observed in other studies, such as the comparative analysis of *Arachis*, *L. japonicus*, and *M. truncatula*, where *Medicago* regions with high synteny to *Arachis* had low retrotransposon density, while regions of low synteny had high transposon densities (Bertioli et al. 2009). Our comparative mapping of two lupin genomic regions (017B07 from linkage group NLL-20 and KB433973 from NLL-17) provided novel evidence for ancient duplications in all of the studied legume species (Fig. 4). For KB433973, we identified whole-region duplications in *G. max* (four copies, found on chromosomes 1, 5, 11, and 17), *P. vulgaris* (two copies, found on chromosomes 2 and 3) and *M. truncatula* (two copies, found on chromosomes 4 and 5), and fragmentary duplications in *L. japonicus* (two copies, found on chromosomes 2 and 4) (Fig. 5). Generally, the collinearity of sequence alignments was almost perfectly preserved among species. In the duplicated segments, however, some rearrangements were observed; these included inversions of syntenic blocks spanning ∼3 kb (e.g., for *P. vulgaris* and *L. japonicus*) to 30 kb (*G. max*) and multiple interspersed insertions of other non-syntenic sequences. One large insertion (23.7 Mb) adjacent to a 644-kb inversion was identified on soybean chromosome 5. The 017B07 region was found to have accumulated more structural modifications than KB433973 during the evolutionary history of the species (Fig. 6). A high level of synteny was noted only for species from the *Phaseolus* clade. In the other two species studied, the synteny was observed for approximately half of the lupin sequence (the first 70 kb revealed collinearity to *Medicago* and the last 60 kb to *Lotus*). Full duplication of the region was identified only in the *G. max* genome (on chromosomes 10 and 20); this was not an exact copy but rather included a 13-Mb insertion and a 340-kb inversion. The soybean genome is considered to be one of the most complex plant genomes currently under investigation, as it has encountered multiple whole-genome duplications, genome diploidization, and chromosomal rearrangements (Shoemaker et al. 2006). Thus, copied DNA sequences exist in the genome. Notably, 61.4 % of the homologous genes are present in two chromosomes and 21.5 % in another four (Schmutz et al. 2010). These segmental duplications are well preserved in the analyzed GRRs. Chromosome 7 of *P. vulgaris* and chromosome 10 of *G. max* retained the entirety of clone 017B07, which underwent structural changes (three inversions and two insertions) that yielded regions of similar length in *Phaseolus* (615 kb) and *Glycine* (607 kb) compared to *Lupinus* (138 kb). Partial duplications with distortions in the structures or orientations of the corresponding sequences were found in all species but *L. japonicus*. Notably, the incomplete copies of 017B07 found on *P. vulgaris* chromosome 8 and *G. max* chromosome 18 show a partial lack of similarity to the *L. angustifolius* sequence but are nearly identical in their lengths and nucleotide positions on the chromosomes. This indicates that the common ancestor of *G. max* and *P. vulgaris* apparently carried a duplication of this segment.

To summarize, the following microsyntenic relationships can be inferred for *L. angustifolius* (La), *L. japonicus* (Lj), *M. truncatula* (Mt), *C. cajan* (Cj), *P. vulgaris* (Pv), and *G. max* (Gm): (a) La17-Lj2-Mt5-Cj6-Pv2-Gm1-Gm11; (b) La17-Mt4-Pv3-Gm5-Gm17; (c) La20-Lj5-Mt1-Cj2-Cjscaffold000310-Cjscaffold000132622-Pv7-Gm10-Gm20; and (d) La20-Mt7-Pv8-Gm18. Due to regularly dispersed local insertions, all of the syntenic regions in the analyzed species were considerably longer than those of *L. angustifolius* (by ∼3-fold in *Cajanus* and *Medicago* and 5- to 6-fold in *Lotus*, *Glycine*, and *Phaseolus*).

### Reference to the Macrosyntenic Relationships

Legume species, including those originating from evolutionarily distant clades, are well known to retain basic synteny in relatively large blocks (several hundred kb to several Mb) localized in the euchromatin (Cannon et al. 2009). For example, two *G. max* BAC clones with high exon densities had homologies with various legume species, including *C. cajan*, *P. vulgaris*, *M. truncatula*, *L. japonicus*, *P. sativum*, *L. albus*, and others (Belarmino et al. 2012). Nevertheless, the ancestral patterns were distorted by extensive chromosome breakages and rearrangements, which probably occurred after the separation of these clades. The development of various genomic resources (e.g., whole-genome sequences, transcriptome sequences, and integrated linkage maps with sequence-tagged loci) has greatly facilitated the comparative mapping of phylogenetically diverse legume plants (Sato et al. 2010). Mapping of the legume anchor markers representing single-copy genes uncovered extensive macrosynteny between *L. japonicus* and *P. vulgaris* for about the half of the linkage groups, as well as large blocks of collinearity between *P. vulgaris* and *M. truncatula*, which also had some limited links to *Arachis* species. The conserved *Phaseolus*–*Medicago* segments were generally shorter than the *Phaseolus*–*Lotus* segments and showed more chromosome rearrangements (Hougaard et al. 2008). Furthermore, a survey based on two *G. max* BAC clones representing a 170-kb GRR (∼11 genes/100 kb) revealed that the collinearity was fragmented between *G. max*, *M. truncatula*, and *A. thaliana* despite the presence of many shared gene loci in the same orders and orientations, suggesting that extensive additions or deletions occurred after the divergence of these species (Schlueter et al. 2008). A homology search involving alignment of the first gene-based *L. angustifolius* linkage map to the *M. truncatula* genome sequence revealed regions of conserved synteny on seven *M. truncatula* chromosomes and 14 *L. angustifolius* chromosomes (Nelson et al. 2006). When the new genetic map of *L. angustifolius* was compared to *L. japonicus* pseudomolecules, 34 segments with conserved collinearity were detected in 17 *L. angustifolius* linkage groups. In three linkage groups, NLL-06, NLL-08, and NLL-12, the locus order was conserved over large regions. However, even these relatively well-conserved regions showed evidence of chromosome translocations and inversions (Nelson et al. 2010).

A study based on the gene-anchored linkage map of *P. vulgaris* revealed that *P. vulgaris*, *L. japonicus*, and *M. truncatula* share distinct macrosyntenic blocks, with dot plots exhibiting a number of relationships, including Lj2-Pv2-Mt5, Lj5-Pv7-Mt1, and Pv8-Mt7 (McConnell et al. 2010). These syntenic links, which were previously identified based on the collinearity of genetic markers, were also found in our present study on microsynteny. Thus, despite the complex evolution of legumes (e.g., whole-genome duplication events followed by extensive chromosome rearrangements leading to a degree of diploidization), some nuclear genome sequences were unaffected by any substantial modification and thus retained their quasi-ancestral structures. It should be noted that, in addition to the major duplication event that occurred early during legume evolution (58 Mya), individual duplication event occurred later in the evolving lineage of *G. max* (Schmutz et al. 2010).

Despite strong similarities in the orders and orientations of some conserved sequences, the genome segments of *P. vulgaris* and *G. max* that showed synteny to the narrow-leafed lupin were approximately twice as long as the corresponding regions in *C. cajan*. The relationships observed between *C. cajan*, *P. vulgaris*, and *G. max* entirely match the phylogenic trees derived from molecular data analyses of legumes. These three species are representatives of the *Phaseolus* clade, which diverged from the IRLC clade (the other main clade of the Papilionoideae) about 54 Mya. The *C. cajan* branch evolved 25.5 Mya, whereas *P. vulgaris* and *G. max* diverged 19 Mya (Lavin et al. 2005; Stefanović et al. 2009). A high degree of collinearity between certain regions of euchromatin in *P. vulgaris* and *G. max* was previously reported: alignment of the *P. vulgaris* genetic map to *G. max* genome sequences revealed the existence of 55 macrosyntenic blocks with a mean physical length of 4.9 Mb (McClean et al. 2010). These blocks included the linkage groups and chromosome links identified in this study (e.g., Pv2-Gm11-Gm11, Pv7-Gm10-Gm20, and Pv18-Gm18). This convergence of results indicates that the synteny between the GRRs of these two legume genomes is maintained at both the gene and chromosome levels. A prior analysis of the structural variation of two *G. max* homologous regions versus those of *P. vulgaris* (Gm8-Gm15-Pv5) revealed strong microcollinearity and high-gene retention rates (Lin et al. 2010). Nevertheless, several structural variations between these regions were determined, such as inversions, translocations, insertions/deletions, and the remnants of nested transposon insertions. The correspondence between *P. vulgaris* and *G. max* chromosomes was also verified by a previous in silico mapping of the phenylpropanoid pathway genes shared by these two species, which confirmed the existence of major syntenic blocks, including Pv2-Gm1-Gm11, Pv3-Gm5-Gm17, Pv7-Gm10-Gm20, and Pv8-Gm18 (Reinprecht et al. 2013). Thus, our present results and the previous findings together show convincingly that these regions are conserved among the main clades of Papilionoideae, as represented by the genistoids (*Lupinus*), dalbergioids (*Arachis*), millettioids (*Cajanus*, *Glycine*, *Phaseolus*), IRLC (*Medicago*), and robinioids (*Lotus*).

## Conclusions

1. The identified *L. angustifolius* GRRs, conserved among five reference legume species representing main Papilionoideae clades, are remnants of the common ancestral genome.
2. The comparative analysis of GRRs provides novel evidence for ancient duplications in five studied legume species (*M. truncatula*, *G. max*, *L. japonicus*, *P. vulgaris*, and *C. cajan*).
3. The BAC clones originating from these GRRs, as universal cytogenetic markers, constitute resources for further legume genomic studies.

## Electronic Supplementary Material

Below is the link to the electronic supplementary material.

Online Resource 1 Functional annotation of BAC-end sequences (PDF 90 kb)

Online Resource 2 Functional annotation of BAC sequences (PDF 36 kb)

Online Resource 3 List of scaffolds anchored in BAC-end sequences (PDF 24 kb)

Online Resource 4 Expansion profiles of repetitive elements in the genomes of model legume species compared to *L. angustifolius* scaffolds. Twelve narrow-leafed lupin repeat-rich scaffolds are shown. Circos plots are ordered in columns according to the species (*Cajanus cajan*, *Glycine max*, *Lotus japonicus*, *Medicago truncatula*, and *Phaseolus vulgaris*). Scaffolds were sorted by the expansiveness of their repetitive elements, and are given from the most ubiquitous to the most specific. Reference legume chromosomes are shown on the left side of the external rings of each plot, while narrow-leafed lupin regions are shown on the right. Annotation data are presented on the internal rings as follows: genes (green), and interspersed repeats (gray). Ribbons symbolize homologous links, as assessed by DNA sequence similarity. (PDF 22,628 kb)

Online Resource 5 Summary of scaffold annotation (PDF 18 kb)

Online Resource 6 Linkage groups of the *L. angustifolius* genetic map supplemented with new genetic markers (PDF 623 kb)

Online Resource 7 Data on the genetic markers developed in this study (PDF 53 kb)

## References

[CR1] Ainouche AK, Bayer RJ (1999). Phylogenetic relationships in Lupinus (Fabaceae: Papilionoideae) based on internal transcribed spacer sequences (ITS) of nuclear ribosomal DNA. Am J Bot.

[CR2] Aïnouche A, Bayer RJ, Misset M-T (2004). Molecular phylogeny, diversification and character evolution in Lupinus (Fabaceae) with special attention to Mediterranean and African lupines. Plant Syst Evol.

[CR3] Belarmino LC, da S Oliveira AR, Brasileiro-Vidal AC (2012). Mining plant genome browsers as a means for efficient connection of physical, genetic and cytogenetic mapping: an example using soybean. Genet Mol Biol.

[CR4] Bertioli DJ, Moretzsohn MC, Madsen LH (2009). An analysis of synteny of Arachis with Lotus and Medicago sheds new light on the structure, stability and evolution of legume genomes. BMC Genomics.

[CR5] Boersma JG, Pallotta M, Li C (2005). Construction of a genetic linkage map using MFLP and identification of molecular markers linked to domestication genes in narrow-leafed lupin (Lupinus angustifolius L.). Cell Mol Biol Lett.

[CR6] Boersma JG, Buirchell BJ, Sivasithamparam K, Yang H (2007). Development of a sequence-specific PCR marker linked to the Ku gene which removes the vernalization requirement in narrow-leafed lupin. Plant Breed.

[CR7] Boersma JG, Buirchell BJ, Sivasithamparam K, Yang H (2007). Development of two sequence-specific PCR markers linked to the le gene that reduces pod shattering in narrow-leafed Lupin (Lupinus angustifolius L.). Genet Mol Biol.

[CR8] Cannon SB, May GD, Jackson SA (2009). Three sequenced legume genomes and many crop species: rich opportunities for translational genomics. Plant Physiol.

[CR9] Cardle L, Ramsay L, Milbourne D (2000). Computational and experimental characterization of physically clustered simple sequence repeats in plants. Genetics.

[CR10] Chen M, Presting G, Barbazuk WB (2002). An integrated physical and genetic map of the rice genome. Plant Cell.

[CR11] Drummond CS, Eastwood RJ, Miotto STS, Hughes CE (2012). Multiple continental radiations and correlates of diversification in Lupinus (Leguminosae): testing for key innovation with incomplete taxon sampling. Syst Biol.

[CR12] Ewing B, Hillier L, Wendl MC, Green P (1998). Base-calling of automated sequencer traces using phred. I. Accuracy assessment. Genome Res.

[CR13] Farrar K, Donnison IS (2007). Construction and screening of BAC libraries made from Brachypodium genomic DNA. Nat Protoc.

[CR14] Findley SD, Cannon S, Varala K (2010). A fluorescence in situ hybridization system for karyotyping soybean. Genetics.

[CR15] Florea L, Hartzell G, Zhang Z (1998). A computer program for aligning a cDNA sequence with a genomic DNA sequence. Genome Res.

[CR16] Fonsêca A, Ferreira J, dos Santos TRB (2010). Cytogenetic map of common bean (Phaseolus vulgaris L.). Chromosom Res.

[CR17] Gao L-L, Hane JK, Kamphuis LG (2011). Development of genomic resources for the narrow-leafed lupin (Lupinus angustifolius): construction of a bacterial artificial chromosome (BAC) library and BAC-end sequencing. BMC Genomics.

[CR18] Hougaard BK, Madsen LH, Sandal N (2008). Legume anchor markers link syntenic regions between Phaseolus vulgaris, Lotus japonicus, Medicago truncatula and Arachis. Genetics.

[CR19] Huang X, Madan A (1999). CAP3: a DNA sequence assembly program. Genome Res.

[CR20] Hughes C, Eastwood R (2006). Island radiation on a continental scale: exceptional rates of plant diversification after uplift of the Andes. Proc Natl Acad Sci U S A.

[CR21] Iseli C, Jongeneel CV, Bucher P (1999) ESTScan: a program for detecting, evaluating, and reconstructing potential coding regions in EST sequences. Int Conf Intell Syst Mol Biol, 138–148 10786296

[CR22] Jurka J, Kapitonov VV, Pavlicek A (2005). Repbase Update, a database of eukaryotic repetitive elements. Cytogenet Genome Res.

[CR23] Kaczmarek A, Naganowska B, Wolko B (2009). Karyotyping of the narrow-leafed lupin (Lupinus angustifolius L.) by using FISH, PRINS and computer measurements of chromosomes. J Appl Genet.

[CR24] Kasprzak A, Safár J, Janda J (2006). The bacterial artificial chromosome (BAC) library of the narrow-leafed lupin (Lupinus angustifolius L.). Cell Mol Biol Lett.

[CR25] Katagiri T, Kidd C, Tomasino E (2005). A BAC-based physical map of the Nile tilapia genome. BMC Genomics.

[CR26] Konieczny A, Ausubel FM (1993). A procedure for mapping Arabidopsis mutations using co-dominant ecotype-specific PCR-based markers. Plant J.

[CR27] Krzywinski M, Schein J, Birol I (2009). Circos: an information aesthetic for comparative genomics. Genome Res.

[CR28] Książkiewicz M, Wyrwa K, Szczepaniak A (2013). Comparative genomics of Lupinus angustifolius gene-rich regions: BAC library exploration, genetic mapping and cytogenetics. BMC Genomics.

[CR29] Lavin M, Herendeen PS, Wojciechowski MF (2005). Evolutionary rates analysis of Leguminosae implicates a rapid diversification of lineages during the tertiary. Syst Biol.

[CR30] Lesniewska K, Ksiazkiewicz M, Nelson MN (2011). Assignment of 3 genetic linkage groups to 3 chromosomes of narrow-leafed lupin. J Hered.

[CR31] Lewis SE, Searle SMJ, Harris N, et al. (2002) Apollo: a sequence annotation editor. Genome Biol 3:RESEARCH0082. doi:10.1186/gb-2002-3-12-research0082 10.1186/gb-2002-3-12-research0082 PMC151184 12537571

[CR32] Li X, Renshaw D, Yang H, Yan G (2010). Development of a co-dominant DNA marker tightly linked to gene tardus conferring reduced pod shattering in narrow-leafed lupin (Lupinus angustifolius L.). Euphytica.

[CR33] Li X, Buirchell B, Yan G, Yang H (2012). A molecular marker linked to the mollis gene conferring soft-seediness for marker-assisted selection applicable to a wide range of crosses in lupin (Lupinus angustifolius L.) breeding. Mol Breed.

[CR34] Lin J-Y, Stupar RM, Hans C (2010). Structural and functional divergence of a 1-Mb duplicated region in the soybean (Glycine max) genome and comparison to an orthologous region from Phaseolus vulgaris. Plant Cell.

[CR35] Lyons E, Pedersen B, Kane J (2008). Finding and comparing syntenic regions among Arabidopsis and the outgroups papaya, poplar, and grape: CoGe with Rosids. Plant Physiol.

[CR36] Manly KF, Robert H, Cudmore J, Meer JM (2001). Map Manager QTX, cross-platform software for genetic mapping. Mamm Genome.

[CR37] Marra MA, Kucaba TA, Dietrich NL (1997). High throughput fingerprint analysis of large-insert clones. Genome Res.

[CR38] McClean PE, Mamidi S, McConnell M (2010). Synteny mapping between common bean and soybean reveals extensive blocks of shared loci. BMC Genomics.

[CR39] McConnell M, Mamidi S, Lee R (2010). Syntenic relationships among legumes revealed using a gene-based genetic linkage map of common bean (Phaseolus vulgaris L.). Theor Appl Genet.

[CR40] Mun J-H, Kim D-J, Choi H-K (2006). Distribution of microsatellites in the genome of Medicago truncatula: a resource of genetic markers that integrate genetic and physical maps. Genetics.

[CR41] Naganowska B, Wolko B, Sliwińska E, Kaczmarek Z (2003). Nuclear DNA content variation and species relationships in the genus Lupinus (Fabaceae). Ann Bot.

[CR42] Neff MM, Turk E, Kalishman M (2002). Web-based primer design for single nucleotide polymorphism analysis. Trends Genet.

[CR43] Nelson MN, Phan HTT, Ellwood SR (2006). The first gene-based map of Lupinus angustifolius L.—location of domestication genes and conserved synteny with Medicago truncatula. Theor Appl Genet.

[CR44] Nelson MN, Moolhuijzen PM, Boersma JG (2010). Aligning a new reference genetic map of Lupinus angustifolius with the genome sequence of the model legume, Lotus japonicus. DNA Res.

[CR45] Ng SHS, Artieri CG, Bosdet IE (2005). A physical map of the genome of Atlantic salmon, Salmo salar. Genomics.

[CR46] O’Rourke JA, Yang SS, Miller SS (2013). An RNA-Seq transcriptome analysis of orthophosphate-deficient white lupin reveals novel insights into phosphorus acclimation in plants. Plant Physiol.

[CR47] Parra-González LB, Aravena-Abarzúa GA, Navarro-Navarro CS (2012). Yellow lupin (Lupinus luteus L.) transcriptome sequencing: molecular marker development and comparative studies. BMC Genomics.

[CR48] Pedrosa A, Sandal N, Stougaard J (2002). Chromosomal map of the model legume Lotus japonicus. Genetics.

[CR49] Ramchiary N, Nguyen VD, Li X (2011). Genic microsatellite markers in Brassica rapa: development, characterization, mapping, and their utility in other cultivated and wild Brassica relatives. DNA Res.

[CR50] Reinprecht Y, Yadegari Z, Perry GE (2013). In silico comparison of genomic regions containing genes coding for enzymes and transcription factors for the phenylpropanoid pathway in Phaseolus vulgaris L. and Glycine max L. Merr. Front. Plant Sci.

[CR51] Revanna KV, Chiu C-C, Bierschank E, Dong Q (2011). GSV: a web-based genome synteny viewer for customized data. BMC Bioinforma.

[CR52] Salamov AA, Solovyev VV (2000). Ab initio gene finding in Drosophila genomic DNA. Genome Res.

[CR53] Sato S, Nakamura Y, Kaneko T (2008). Genome structure of the legume, Lotus japonicus. DNA Res.

[CR54] Sato S, Isobe S, Tabata S (2010). Structural analyses of the genomes in legumes. Curr Opin Plant Biol.

[CR55] Schlueter JA, Scheffler BE, Jackson S, Shoemaker RC (2008). Fractionation of synteny in a genomic region containing tandemly duplicated genes across Glycine max, Medicago truncatula, and Arabidopsis thaliana. J Hered.

[CR56] Schmutz J, Cannon SB, Schlueter J (2010). Genome sequence of the palaeopolyploid soybean. Nature.

[CR57] Shah MM, Hassan A (2005). Distribution of genes and recombination on wheat homoeologous group 6 chromosomes: a synthesis of available information. Mol Breed.

[CR58] Shoemaker RC, Schlueter J, Doyle JJ (2006). Paleopolyploidy and gene duplication in soybean and other legumes. Curr Opin Plant Biol.

[CR59] Soderlund C, Longden I, Mott R (1997). FPC: a system for building contigs from restriction fingerprinted clones. Comput Appl Biosci.

[CR60] Stanke M, Morgenstern B (2005). AUGUSTUS: a web server for gene prediction in eukaryotes that allows user-defined constraints. Nucleic Acids Res.

[CR61] Stefanović S, Pfeil BE, Palmer JD, Doyle JJ (2009). Relationships among phaseoloid legumes based on sequences from eight chloroplast regions. Syst Bot.

[CR62] Sulston J, Mallett F, Durbin R, Horsnell T (1989). Image analysis of restriction enzyme fingerprint autoradiograms. Comput Appl Biosci.

[CR63] Sweetingham MW, Yang H, Buirchell BJ Shea G, Shield I (2005) Resistance to rust in narrow-leafed lupin and development of molecular markers. In: van Santen E, Hill GD (eds) México, where old and new world lupins meet. Proceedings of the 11th International Lupin Conference, Guadalajara, Jalisco, Mexico, 4–9 May 2006, pp 14–16

[CR64] Temnykh S, DeClerck G, Lukashova A (2001). Computational and experimental analysis of microsatellites in rice (Oryza sativa L.): frequency, length variation, transposon associations, and genetic marker potential. Genome Res.

[CR65] Untergasser A, Nijveen H, Rao X (2007). Primer3Plus, an enhanced web interface to Primer3. Nucleic Acids Res.

[CR66] Varshney RK, Chen W, Li Y (2012). Draft genome sequence of pigeonpea (Cajanus cajan), an orphan legume crop of resource-poor farmers. Nat Biotechnol.

[CR67] Voorrips RE (2002). MapChart: software for the graphical presentation of linkage maps and QTLs. J Hered.

[CR68] Xu Z, Kohel RJ, Song G (2008). An integrated genetic and physical map of homologous chromosomes 12 and 26 in upland cotton (G. hirsutum L.). BMC Genomics.

[CR69] Yang H, Sweetingham MW, Cowling WA, Smith PMC (2001). DNA fingerprinting based on microsatellite-anchored fragment length polymorphisms, and isolation of sequence-specific PCR markers in lupin (Lupinus angustifolius L.). Mol Breed.

[CR70] Yang H, Shankar M, Buirchell J (2002). Development of molecular markers using MFLP linked to a gene conferring resistance to Diaporthe toxica in narrow-leafed lupin ( Lupinus angustifolius L.). Theor Appl Genet.

[CR71] Yang H, Boersma JG, You M (2004). Development and implementation of a sequence-specific PCR marker linked to a gene conferring resistance to anthracnose disease in narrow-leafed lupin (Lupinus angustifolius L.). Mol Breed.

[CR72] Yang H, Renshaw D, Thomas G (2008). A strategy to develop molecular markers applicable to a wide range of crosses for marker assisted selection in plant breeding: a case study on anthracnose disease resistance in lupin (Lupinus angustifolius L.). Mol Breed.

[CR73] Yang H, Tao Y, Zheng Z (2012). Application of next-generation sequencing for rapid marker development in molecular plant breeding: a case study on anthracnose disease resistance in Lupinus angustifolius L. BMC Genomics.

[CR74] Yang H, Tao Y, Zheng Z (2013). Rapid development of molecular markers by next-generation sequencing linked to a gene conferring phomopsis stem blight disease resistance for marker-assisted selection in lupin (Lupinus angustifolius L.) breeding. Theor Appl Genet.

[CR75] Yang H, Tao Y, Zheng Z (2013). Draft genome sequence, and a sequence-defined genetic linkage map of the legume crop species Lupinus angustifolius L. PLoS One.

[CR76] You M, Boersma JG, Buirchell BJ (2005). A PCR-based molecular marker applicable for marker-assisted selection for anthracnose disease resistance in lupin breeding. Cell Mol Biol Lett.

[CR77] Young ND, Debellé F, Oldroyd GED (2011). The Medicago genome provides insight into the evolution of rhizobial symbioses. Nature.

[CR78] Zielezinski A, Potarzycki P, Książkiewicz M, Karłowski W (2012). Annotating a non-model plant genome—a study on the narrow-leafed lupin. Biotechnologia.

