# Supplementary material for: Remnants of the Legume Ancestral Genome Preserved in Gene-Rich Regions: Insights from Lupinus angustifolius Physical, Genetic, and Comparative Mapping
Source: Plant Mol Biol Report. 2014 May 15;33(1):84–101. doi: 10.1007/s11105-014-0730-4 (PMC4295026; doi:10.1007/s11105-014-0730-4)
Supplement: Supplementary file 2 — Functional annotation of BAC sequences (PDF 36 kb) [file 11105_2014_730_MOESM2_ESM.pdf]

Functional annotation of BAC sequences

BAC clone, accession	Predicted gene	Repetitive elements	*Lupinus albus* EST coverage	*Lupinus luteus* EST coverage	EST coverage	Unigene coverage	Gene / protein name	Reference accession (excluding repetitive elements)	Protein coverage (excluding repetitive elements)	Gene start	Gene end	Strand	Exon coordinates	CDS coordinates
017B07 HF937076	1	-	100%	100%	100%	100%	callose synthase	Q9SFU6	protein fragment ~ 4%	1	1731	C	366..570;1307..1731;	366..567;
	2	-	100%	100%	37%	94%	callose synthase	Q9SFU6	protein fragment ~ 9%	3495	5751	C	3495..3978;4235..4328;4401..4456;4810..4910;5353..5751;	3730..3978;4235..4328;4401..4456;4810..4910;5353..5536;
	3	-	100%	100%	100%	100%	PRLI-interacting factor A	XM_003592766	complete protein	6646	9199	W	6646..9199;	6926..8503;
	4	-	87%	99%	90%	92%	plastid-lipid-associated protein 12	XM_003556512	complete protein	10695	14693	W	10695..10907;10985..11038;11438..11512;12105..12224;13004..13195;13344..13427;13511..13603;13761..13814;13898..13977;14355..14462;14621..14693	10695..10907;10985..11038;11438..11512;12105..12224;13004..13195;13344..13427;13511..13603;13761..13814;13898..13975;14356..14460;14622..14693
	5	-	94%	99%	97%	100%	uncharacterized protein	XM_003592764	protein fragment ~ 36%	14803	20841	C	15034..15234;15319..15954;16220..16289;20142..20155	15034..15234;15319..15954;16220..16288;20144..20155
	6	-	62%	100%	95%	97%	CDP-diacylglycerol-glycerol-3-phosphate 3-phosphatidyltransferase	XM_003592761	protein fragment ~ 65%	16974	22706	W	16974..16983;20110..20496;20682..20767;20894..20944;21075..21188;22075..22131;22218..22303;22586..22706	16974..16982;20112..20495;20684..20767;20894..20944;21075..21188;22075..22131;22218..22301;22587..22706
	gap	-	-	-	-	-	-	-	-	-	-	-	26554_26653	-
	7	-	98%	100%	97%	100%	casein kinase I	XM_003620965	complete protein	26566	31006	W	26566..26728;26976..27086;27195..27268;27382..27451;27540..27595;27742..27813;28015..28109;28194..28260;28410..28539;28625..28840;28950..29080;29185..29359;29440..29530;30149..30306;30426..30614;30699..31006;	26657..26728;26976..27086;27195..27268;27382..27451;27540..27595;27742..27813;28015..28109;28194..28260;28410..28539;28625..28840;28950..29080;29185..29359;29440..29530;30149..30306;30426..30614;30699..30758;
	8	-	100%	100%	100%	100%	uncharacterized protein	XM_003592757	complete protein	32826	35821	W	32826..32995;33145..33277;33624..33712;33817..33936;34869..35002;35138..35163;35243..35399;35538..35821;	32909..32995;33145..33277;33624..33712;33817..33936;34869..35002;35138..35163;35243..35399;35538..35589;
	9	-	98%	98%	73%	83%	probable protein phosphatase 2C	Q9LHJ9	protein fragment ~ 59%	39296	40600	W	39296..39708;39926..40333;40459..40600;	39391..39708;39926..40333;40459..40539;
	gap	-	-	-	-	-	-	-	-	-	-	-	40598_40697	-
	10	-	83%	100%	100%	99%	high mobility group B protein 15	Q9MAT6	protein fragment ~ 76%	41696	47717	W	41696..41728;44627..44839;45284..45362;45863..46028;46119..46214;46672..47066;47156..47717;	44661..44839;45284..45362;45863..46028;46119..46214;46672..47066;47156..47458;
	11	-	100%	100%	100%	97%	high mobility group B protein 7	Q8LDF9	protein fragment ~ 50%	48296	50495	W	48296..48458;48592..48674;48772..48817;48925..49038;49124..49181;49293..49337;49427..49519;49600..50495;	48352..48458;48592..48674;48772..48817;48925..49038;49124..49181;49293..49337;49427..49519;49600..49674;
	12	Gypsy	45%	99%	83%	97%	predicted: protease 2-like	AK287419	complete protein	52041	60121	C	52041..52326;52699..52822;52945..53026;53094..53159;53889..54254;54699..54857;55901..56112;56572..56600;56970..57316;57434..57544;57660..57854;57963..58246;59643..60121;	52236..52326;52699..52822;52945..53026;53094..53159;53889..54254;54699..54857;55901..56112;56572..56600;56970..57316;57434..57544;57660..57854;57963..58246;59643..59982;
	13	-	88%	100%	100%	100%	GEM-like protein	Q9FMW4	complete protein	61222	62951	C	61222..61495;61696..61970;62254..62334;62424..62951;	61322..61495;61696..61970;62254..62334;62424..62559;
	14	-	100%	100%	96%	96%	uncharacterized protein	FG093169	complete protein	65157	66841	W	65157..65222;65344..65397;65848..65970;66704..66841	65157..65222;65344..65397;65848..65970;66704..66841
	15	RTE, SINE	100%	100%	48%	48%	Ras-related protein	BT097872.1	complete protein	71624	74731	W	71624..71626;71730..71849;71946..72013;72988..73096;73187..73255;73408..73465;73917..74152;74515..74566;74664..74731	71624..71626;71730..71849;71946..72011;72989..73096;73187..73255;73408..73464;73919..74152;74515..74565;74666..74731
	16	-	53%	100%	98%	100%	uncharacterized protein	XM_003552634	complete protein	81503	84491	C	81503..81829;81972..82173;82260..82328;82426..83811;84106..84491;	81759..81829;81972..82173;82260..82328;82426..83811;84106..84108;
	17	-	76%	100%	100%	100%	zinc finger protein	Q9SLD4	protein fragment ~ 75%	87026	87982	W	87026..87982;	87172..87693;
	18	-	100%	85%	73%	73%	uncharacterized protein	BW650777	complete protein	92938	94151	C	92938..93723;93790..93875;94069..94151;	93619..93723;93790..93867;
	19	-	99%	100%	93%	100%	uncharacterized protein	XM_003556073	complete protein	95536	98332	W	95536..95552;96525..96615;96711..97205;97406..97461;97545..98332;	96558..96615;96711..97205;97406..97461;97545..97730;
	20	-	42%	57%	63%	97%	uncharacterized protein	XM_003555138	complete protein	98655	99685	C	98655..99228;99331..99400;99505..99685	98655..99227;99333..99398;99506..99685
	21	-	98%	100%	92%	100%	ABC transporter B family	Q8LPT1	protein fragment ~ 78% (end of BAC clone)	101683	109008	C	101683..103370;104342..104583;104664..105451;105740..105839;105913..106081;106180..106400;107165..107475;107555..107814;108952..109008;	102141..103370;104342..104583;104664..105451;105740..105839;105913..106081;106180..106400;107165..107475;107555..107814;108952..109008;
075D16 HF937080	gap	-	-	-	-	-	-	-	-	-	-	-	1075_1174	-
	1	many repeats	88%	82%	87%	13%	putative AC transposase (repetitive element)	-	-	15398	21261	C	15398..15805;15893..15942;16090..16181;16302..16980;21104..21261;	15520..15805;15893..15942;16090..16181;16302..16959;
	2	RTE1_MT	23%	38%	17%	-	-	-	-	21946	24562	W	21946..22082;22506..22648;24309..24562;	21972..22082;22506..22648;24309..24474;
	gap	-	-	-	-	-	-	-	-	-	-	-	25064_25163	-
	gap	-	-	-	-	-	-	-	-	-	-	-	41633_41732	-
	3	Copia	100%	100%	-	-	-	-	-	42214	43941	C	42214..43941;	42568..43833;
	4	Copia	100%	100%	19%	14%	retrovirus-related Pol polyprotein from transposon (repetitive element)	-	-	44360	49981	C	44360..44888;45108..45401;45558..46419;47322..47574;48109..49424;49884..49981;	44495..44888;45108..45401;45558..46419;47322..47574;48109..49368;
	gap	-	-	-	-	-	-	-	-	-	-	-	49531_49630	-
	gap	-	-	-	-	-	-	-	-	-	-	-	50290_50389	-
	5	Gypsy, Copia	100%	28%	-	-	-	-	-	52362	59291	C	52362..53031;53985..54409;54676..55047;59228..59291;	52550..53031;53985..54409;54676..54929;
	gap	-	-	-	-	-	-	-	-	-	-	-	64642_64741	-
	6	Copia	96%	96%	-	-	-	-	-	65124	66761	C	65124..66761;	65396..66667;
	7	Copia	99%	100%	24%	30%	retrovirus-related Pol polyprotein from transposon (repetitive element)	-	-	67087	72261	C	67087..69450;70066..70262;70347..70446;70984..72261;	67246..69450;70066..70262;70347..70446;70984..72120;
	gap	-	-	-	-	-	-	-	-	-	-	-	72187_72286	-
	8	TORTL1, Copia	81%	81%	-	-	-	-	-	76508	80541	C	76508..77018;77106..77238;77435..77505;77652..77897;78536..78637;80500..80541;	76767..77018;77106..77238;77435..77505;77652..77897;78536..78634;
	9	Copia and others	100%	96%	51%	40%	retrovirus-related Pol polyprotein from transposon (repetitive element)	-	-	82706	87776	W	82706..82783;83421..83661;83788..84135;84652..84904;86335..86684;86827..87776;	83449..83661;83788..84135;84652..84904;86335..86684;86827..87711;
	10	Copia	100%	97%	-	-	-	-	-	88196	90090	W	88196..90090;	88302..89579;
	11	-	100%	100%	100%	100%	heme oxygenase 1, chloroplastic	O48782.2	protein fragment ~ 50% (end of BAC clone)	95468	98086	C	95468..95802;96175..96282;97352..97575;	95710..95802;96175..96282;97352..97575;
112N18 HF937077	1	-	95%	99%	70%	90%	pleiotropic drug resistance protein 1	Q76CU2.1	protein fragment 11% (end of BAC clone)	1	2601	C	159..247;329..449;2317..2601;	159..247;329..449;2317..2573;
	gap	-	-	-	-	-	-	-	-	-	-	-	5426_5525	-
	2	-	99%	95%	-	-	uncharacterized protein	-	-	10931	12321	C	10931..12155;12264..12321;	11796..12155;12264..12266;
	gap	-	-	-	-	-	-	-	-	-	-	-	12805_12904	-
	3	Gypsy	97%	98%	17%	19%	-	-	-	18486	23451	W	18486..19166;19245..19844;19968..20194;20487..20725;21096..21848;22374..23002;23063..23451;	18692..19166;19245..19844;19968..20194;20487..20725;21096..21848;22374..23002;23063..23331;
	4	Gypsy	93%	91%	-	-	-	-	-	25016	26751	C	25016..26131;26381..26751;	25542..26131;26381..26534;
	5	Gypsy	-	-	-	-	-	-	-	28076	28660	W	28076..28660;	28158..28433;
	6	-	80%	88%	87%	87%	ethanolamine kinase A-like	XM_003522856	protein fragment 40% (end of BAC clone)	41575	43789	C	41575..42120;42206..42298;43403..43469;43549..43654;43724..43789;	41992..42120;42206..42298;43403..43469;43549..43654;43724..43787;
119M23 HF937078	1	EnSpm	80%	57%	16%	26%	-	-	-	1	8559	W	66..220;816..870;952..1017;1352..1524;1630..1740;2003..2095;2350..2396;2673..2879;2963..3055;3153..3224;4190..4588;8278..8559;	66..220;816..870;952..1017;1352..1524;1630..1740;2003..2095;2350..2396;2673..2879;2963..3055;3153..3224;4190..4408;
	2	Copia	100%	82%	-	-	-	-	-	9816	11624	W	9816..10142;10245..10463;10743..11624;	10005..10142;10245..10463;10743..11279;
	3	Copia and others	100%	96%	9%	9%	-	-	-	13596	18923	W	13596..13965;14121..14362;15641..15914;16294..16635;18487..18923;	13789..13965;14121..14362;15641..15914;16294..16635;18487..18795;
	4	Copia	100%	100%	76%	59%	-	-	-	20136	21754	W	20136..20528;20823..21754;	20868..21608;
	5	Copia	95%	87%	27%	15%	gag-pol polyprotein (repetitive element)	-	-	22899	26921	C	22899..23215;24970..25034;25689..26268;26413..26921;	24981..25034;25689..26268;26413..26813;
	6	Copia	100%	100%	57%		retrovirus-related Pol polyprotein from transposon (repetitive element)	-	-	29556	32045	W	29556..30835;30992..32045;	29754..30835;30992..31898;
	7	Copia and others	100%	100%	-	-	-	-	-	32206	38327	W	32206..34000;36814..37072;37760..37879;38114..38327;	32368..33543;
	8	Copia and others	84%	72%	-	-	-	-	-	38446	39986	W	38446..38825;39074..39177;39234..39660;39761..39986;	38529..38825;39074..39177;39234..39660;39761..39874;
	9	Copia	99%	99%	78%	64%	retrovirus-related Pol polyprotein from transposon (repetitive element)	-	-	40736	42518	W	40736..41110;41192..42518;	40973..41110;41192..42289;
	10	Copia	100%	98%	-	-	-	-	-	43196	44983	W	43196..43475;43669..44983;	43317..43475;43669..44520;
	11	Copia	98%	-	-	-	-	-	-	46776	47262	W	46776..47262;	46829..47113;
	12	Copia	91%	-	-	-	-	-	-	51746	52460	W	51746..52460;	51854..52033;
	13	Copia	100%	100%	-	-	-	-	-	54401	55971	C	54401..55026;55108..55165;55268..55971;	54708..55026;55108..55165;55268..55775;
	14	Copia	99%	91%	30%	-	-	-	-	61226	62596	W	61226..61279;61413..61628;61706..62596;	61416..61628;61706..62149;
	15	-	-	-	-	-	-	-	-	77876	79096	W	77876..79096;	78082..78450;
	16	Copia	98%	87%	14%	-	retrovirus-related Pol polyprotein from transposon (repetitive element)	-	-	81707	87361	C	81707..82457;83907..84005;84542..84754;84862..84912;85019..85156;85695..85798;86021..86592;87269..87361;	82102..82457;83907..84005;84542..84754;84862..84912;85019..85156;85695..85798;86021..86529;
	17	Copia	-	-	48%	-	-	-	-	88036	90492	W	88036..88379;89290..90492;	88165..88379;89290..89956;
	gap	-	-	-	-	-	-	-	-	-	-	-	89810_89909	-
	gap	-	-	-	-	-	-	-	-	-	-	-	95940_96039	-
136B16 HF937079	1	-	-	63%	63%	63%	probable ethanolamine kinase	XM_003527108	protein fragment ~ 12% (AA 1-47)	11916	13368	W	11916..12210;12289..13368;	12074..12210;12289..12370;
	2	Copia	46%	-	-	-	-	-	-	15646	18000	W	15646..15783;17046..17129;17295..18000;	17117..17129;17295..17731;
	3	Copia	100%	97%	11%	-	retrovirus-related Pol polyprotein from transposon (repetitive element)	-	-	18086	22941	W	18086..19985;20073..20199;20288..20385;20922..21671;21825..22941;	18185..19985;20073..20199;20288..20385;20922..21671;21825..22783;
	4	-	-	76%	26%	26%	probable ethanolamine kinase	XM_003527108	protein fragment ~ 24% (AA 129-219)	23856	26179	W	23856..24083;24242..24402;24489..24589;24858..25069;25434..25556;25883..25974;26085..26179;	24005..24083;24242..24402;24489..24589;24858..25069;25434..25556;25883..25974;26085..26159;
	gap	-	-	-	-	-	-	-	-	-	-	-	26398_26497	-
	5	Gypsy	87%	91%	5%	10%	RNA-directed DNA polymerase homolog (repetitive element)	-	-	29656	34366	W	29656..30294;30904..31094;31186..31417;31507..31582;31712..32018;32317..32687;33450..33623;33692..34366;	29789..30294;30904..31094;31186..31417;31507..31582;31712..32018;32317..32687;33450..33623;33692..34180;
	6	-	91%	100%	100%	100%	probable ethanolamine kinase	XM_003527108	protein fragment ~ (AA 219-378)	47041	50311	C	47041..47586;47672..47764;48869..48935;49015..49120;49651..49735;49866..50311;	47458..47586;47672..47764;48869..48935;49015..49120;49651..49735;49866..49871;
	7	Gypsy	97%	84%	12%	11%	RNA-directed DNA polymerase homolog (repetitive element)	-	-	50706	54811	W	50706..51062;51290..52035;52176..52286;52389..52607;52703..53198;53307..53379;53457..53507;53640..53763;54027..54135;54514..54811;	50780..51062;51290..52035;52176..52286;52389..52607;52703..53198;53307..53379;53457..53507;53640..53763;54027..54135;54514..54683;
	gap	-	-	-	-	-	-	-	-	-	-	-	56654_56753	-
	8	-	99%	99%	96%	96%	serine-threonine kinase receptor-associated protein-like	XM_003524478	protein fragment ~ 75%	59622	65800	C	59622..59884;60200..60339;60418..60555;60680..60855;61206..61287;65040..65175;65683..65800	59622..59882;60201..60338;60420..60554;60682..60855;61206..61286;65042..65173;65684..65800
	9	-	60%	-	-	-	-	-	-	69706	70234	W	69706..70234;	69781..70134;
	10	-	-	-	-	-	-	-	-	70828	71501	C	70828..71501;	70882..71235;
	11	Copia	100%	100%	52%	-	retrovirus-related Pol polyprotein from transposon (repetitive element)	-	-	74226	75442	W	74226..74419;74623..74718;74865..75442;	74270..74419;74623..74718;74865..75269;
	12	-	86%	100%	100%	100%	uncharacterized protein	XP_001762155.1	complete protein	78661	80451	C	78661..80116;80379..80451;	79084..80076;
	13	Copia	95%	95%	15%	18%	retrovirus-related Pol polyprotein from transposon (repetitive element)	-	-	84446	88600	W	84446..85171;85242..85970;86055..86133;86217..86981;87067..87128;87204..88600;	84701..85171;85242..85970;86055..86133;86217..86981;87067..87128;87204..88520;
	gap	-	-	-	-	-	-	-	-	-	-	-	89232_89331	-
	14	-	100%	100%	100%	90%	probable histone H2A	Q9C944.1	complete protein	99265	101168	W	99265..99372;100872..101168;	99265..99372;100872..101168;
	15	-	-	-	-	-	-	-	-	102503	103511	C	102503..102798;102948..103318;103464..103511;	102675..102798;102948..103228;
