# Supplementary material for: Remnants of the Legume Ancestral Genome Preserved in Gene-Rich Regions: Insights from Lupinus angustifolius Physical, Genetic, and Comparative Mapping
Source: Plant Mol Biol Report. 2014 May 15;33(1):84–101. doi: 10.1007/s11105-014-0730-4 (PMC4295026; doi:10.1007/s11105-014-0730-4)
Supplement: Supplementary file 3 — List of scaffolds anchored in BAC-end sequences (PDF 24 kb) [file 11105_2014_730_MOESM3_ESM.pdf]

List of scaffolds anchored in BAC-end sequences

Scaffold Accession	Matching BAC-end sequence	Scaffold length (nt)	Total repeat length in scaffold (nt)	Total repeat coverage in scaffold (%)	Linkage group assignment	Type of BAC-FISH signal
AOCW01001143.1	016J01_5	1116	57	5.11		r
AOCW01003937.1	101K10_3, 140K16_3	1827	0	0.00		r
AOCW01003955.1	064M08_5	2346	535	22.80		r
AOCW01008005.1	112E01_5	1156	0	0.00	NLL-16	s
AOCW01009949.1	024F15_5	1881	27	1.44		-
AOCW01011149.1	048P24_5	1307	0	0.00		r
AOCW01011161.1	113D15_5	8812	1683	19.10		-
AOCW01012604.1	100A19_5	6217	1185	19.06		-
AOCW01012784.1	128I22_3	3637	0	0.00		r
AOCW01013388.1	064B20_3	12030	7300	60.68		r
AOCW01013809.1	065A19_5	6547	3063	46.78		r
AOCW01015770.1	120E23_5	2812	259	9.21	NLL-02	r
AOCW01015920.1	015P08_3	6854	2383	34.77	NLL-09	s
AOCW01017028.1	140A12_5	6241	569	9.12		r
AOCW01017202.1	015P22_3	803	735	91.53		-
AOCW01021042.1	075D16_3	8265	542	6.56	NLL-03	r
AOCW01021309.1	024B13_5	7386	1053	14.26		r
AOCW01022509.1	072A20_3	2913	658	22.59		r
AOCW01025033.1	109D22_3	4001	1692	42.29	NLL-02	r
AOCW01027015.1	084P14_5	4771	2115	44.33	NLL-08	r
AOCW01027017.1	024B21_3	3821	1192	31.20		-
AOCW01027168.1	026D07_3	2826	340	12.03		r
AOCW01029632.1	072B21_5	1396	0	0.00		r
AOCW01030215.1	024B20_5	4083	2169	53.12		r
AOCW01030300.1	025M16_3	4818	56	1.16		-
AOCW01033531.1	119M19_5	5241	2581	49.25		r
AOCW01033971.1	015P23_5	5046	126	2.50		-
AOCW01034468.1	051C12_5	660	17	2.58	NLL-07	r
AOCW01035391.1	110J23_3	4109	1699	41.35		r
AOCW01041827.1	016M01_5	8045	2827	35.14		r
AOCW01043145.1	096O20_3	18249	1269	6.95		-
AOCW01043673.1	080M14_5	7640	577	7.55		r
AOCW01050396.1	119M23_3	1367	1243	90.93		r
AOCW01051944.1	044I16_5, 064M08_3	19643	4288	21.83		r
AOCW01053116.1	015P23_3	803	0	0.00		-
AOCW01054002.1	112E01_3	9832	4438	45.14	NLL-16	s
AOCW01056629.1	051P10_3	9035	458	5.07	NLL-19	r
AOCW01059436.1	136K12_5	4045	50	1.24		-
AOCW01059754.1	065A19_3	10503	3665	34.89		r
AOCW01063745.1	113G22_3	7020	496	7.07		r
AOCW01064589.1	105I24_5	6332	5341	84.35	NLL-03	r
AOCW01065369.1	064I01_5	1466	227	15.48		r
AOCW01067233.1	035I17_5, 109D22_5, 140A12_3	3998	480	12.01	NLL-02	r
AOCW01068218.1	072O21_5	14631	907	6.20	NLL-16	s
AOCW01075183.1	131L24_3	27043	4751	17.57		r
AOCW01077172.1	037D21_3	10915	102	0.93	NLL-07	r
AOCW01078327.1	043B20_3	4062	1410	34.71		r
AOCW01080598.1	064H23_5	925	353	38.16		r
AOCW01081845.1	111L22_3, 141C03_3	6739	1408	20.89	NLL-17	r
AOCW01082220.1	043F03_3	7418	4026	54.27	NLL-12	r
AOCW01083549.1	136B16_5	12979	195	1.50	NLL-14	r
AOCW01083550.1	136B15_3	2204	96	4.36		-
AOCW01085962.1	080K03_5	11289	3963	35.10	NLL-12	r
AOCW01087314.1	080F20_5	6561	2779	42.36		r
AOCW01093605.1	015G12_5	8854	2053	23.19		-
AOCW01094374.1	024F12_5	8337	1202	14.42	NLL-17	r
AOCW01094963.1	112N18_5	11902	5343	44.89	NLL-14	r
AOCW01095253.1	039C01_5	5333	2805	52.60		r
AOCW01099197.1	064J10_3	11194	1700	15.19		r
AOCW01099691.1	008A03_3	2222	1545	69.53		s
AOCW01103092.1	075D16_5	9368	28	0.30	NLL-03	r
AOCW01105587.1	103A04_5	8205	3755	45.76		r
AOCW01106806.1	024B13_3	1912	37	1.94		r
AOCW01107513.1	026D07_5	7530	2913	38.69		r
AOCW01108721.1	044O23_5	5974	779	13.04		r
AOCW01109785.1	043B20_5	9480	5526	58.29		r
AOCW01110903.1	096M15_5	3149	1604	50.94		-
AOCW01111548.1	105I24_3	11081	958	8.65	NLL-03	r
AOCW01112200.1	064H23_3	27734	11583	41.76		r
AOCW01112223.1	037B11_5	1182	172	14.55		r
AOCW01113580.1	037D21_5	5143	332	6.46	NLL-07	r
AOCW01113596.1	015L10_3	24451	9202	37.63	NLL-17	r
AOCW01113599.1	111L22_5, 141C03_5	9834	594	6.04	NLL-17	r
AOCW01114366.1	119M13_5	673	0	0.00	NLL-04	r
AOCW01117096.1	122H24_3	9352	202	2.16		-
AOCW01119372.1	017B07_5	35303	493	1.50	NLL-20	s
AOCW01123452.1	069K20_5	4193	54	1.29		r
AOCW01123945.1	119M19_3	4284	424	9.90		r
AOCW01124565.1	051N11_5	3753	39	1.04		r
AOCW01125026.1	060B20_5	9402	1146	12.19	NLL-12	r
AOCW01127795.1	072A21_3	12633	5769	45.67		r
AOCW01127831.1	115G22_3	1746	542	31.04		s
AOCW01128973.1	045B15_3	19422	6277	32.32		-
AOCW01129694.1	044O23_3	4400	84	1.91		r
AOCW01131016.1	015P08_5	6430	4085	63.53	NLL-09	s
AOCW01132316.1	107M16_5	6708	315	4.70	NLL-17	r
AOCW01132893.1	017B07_3	32759	634	1.94	NLL-20	s
AOCW01134196.1	096O20_5	20160	2577	12.78		-
AOCW01135960.1	107M16_3	21248	171	0.80	NLL-17	r
AOCW01137563.1	112N18_3	3858	0	0.00	NLL-14	r
AOCW01138041.1	115C21_3	14879	4760	31.99	NLL-14	s
AOCW01145850.1	025C18_3, 123A20_5	11227	5071	45.17		s
AOCW01146884.1	044J16_3	3923	0	0.00		s
AOCW01151017.1	043F03_5	15378	426	2.77	NLL-12	r
AOCW01151108.1	065D15_3	7776	144	1.85		-
AOCW01157525.1	103A04_3	6470	4092	63.25		r
AOCW01157990.1	068O17_5	2883	0	0.00		r
AOCW01161591.1	080M14_3	5786	2520	43.55		r
AOCW01167898.1	016M01_3	646	0	0.00		r
AOCW01169091.1	085L20_3	1213	0	0.00		-
AOCW01170537.1	123A20_3	7219	70	0.97		s
AOCW01170605.1	131C21_3	8796	135	1.53	NLL-01	r
AOCW01172734.1	051P10_5	1345	628	46.69	NLL-19	r
AOCW01172945.1	101K10_5	3110	1355	43.57		r
AOCW01176048.1	080F20_3	2693	113	4.20		r
AOCW01176789.1	110J23_5	1057	1052	99.53		r
AOCW01180219.1	024F15_3	19742	7433	37.65		-
AOCW01182248.1	024F17_3	2834	615	21.70		-
AOCW01185087.1	064B20_5	7128	5350	75.06		r
AOCW01185232.1	016N01_5	678	620	91.45		r
AOCW01185657.1	128I22_5	11367	4354	38.30		r
AOCW01186179.1	072A21_5	2537	1522	59.99		r
AOCW01188466.1	072A20_5	4064	1549	38.12		r
AOCW01190175.1	080K03_3	1797	0	0.00	NLL-12	r

s - single locus BAC-FISH signal (tagging one chromosome pair)
r - repetitive BAC-FISH signal (dispersed over numerous chromosomes)
- - not analyzed
