# Supplementary material for: Remnants of the Legume Ancestral Genome Preserved in Gene-Rich Regions: Insights from Lupinus angustifolius Physical, Genetic, and Comparative Mapping
Source: Plant Mol Biol Report. 2014 May 15;33(1):84–101. doi: 10.1007/s11105-014-0730-4 (PMC4295026; doi:10.1007/s11105-014-0730-4)
Supplement: Supplementary file 4 — Expansion profiles of repetitive elements in the genomes of model legume species compared to L. angustifolius scaffolds. Twelve narrow-leafed lupin repeat-rich scaffolds are shown. Circos plots are ordered in columns according to the species (Cajanus cajan, Glycine max, Lotus japonicus, Medicago truncatula, and Phaseolus vulgaris). Scaffolds were sorted by the expansiveness of their repetitive elements, and are given from the most ubiquitous to the most specific. Reference legume chromosomes are shown on the left side of the external rings of each plot, while narrow-leafed lupin regions are shown on the right. Annotation data are presented on the internal rings as follows: genes (green), and interspersed repeats (gray). Ribbons symbolize homologous links, as assessed by DNA sequence similarity. (PDF 22,628 kb) [file 11105_2014_730_MOESM4_ESM.pdf]

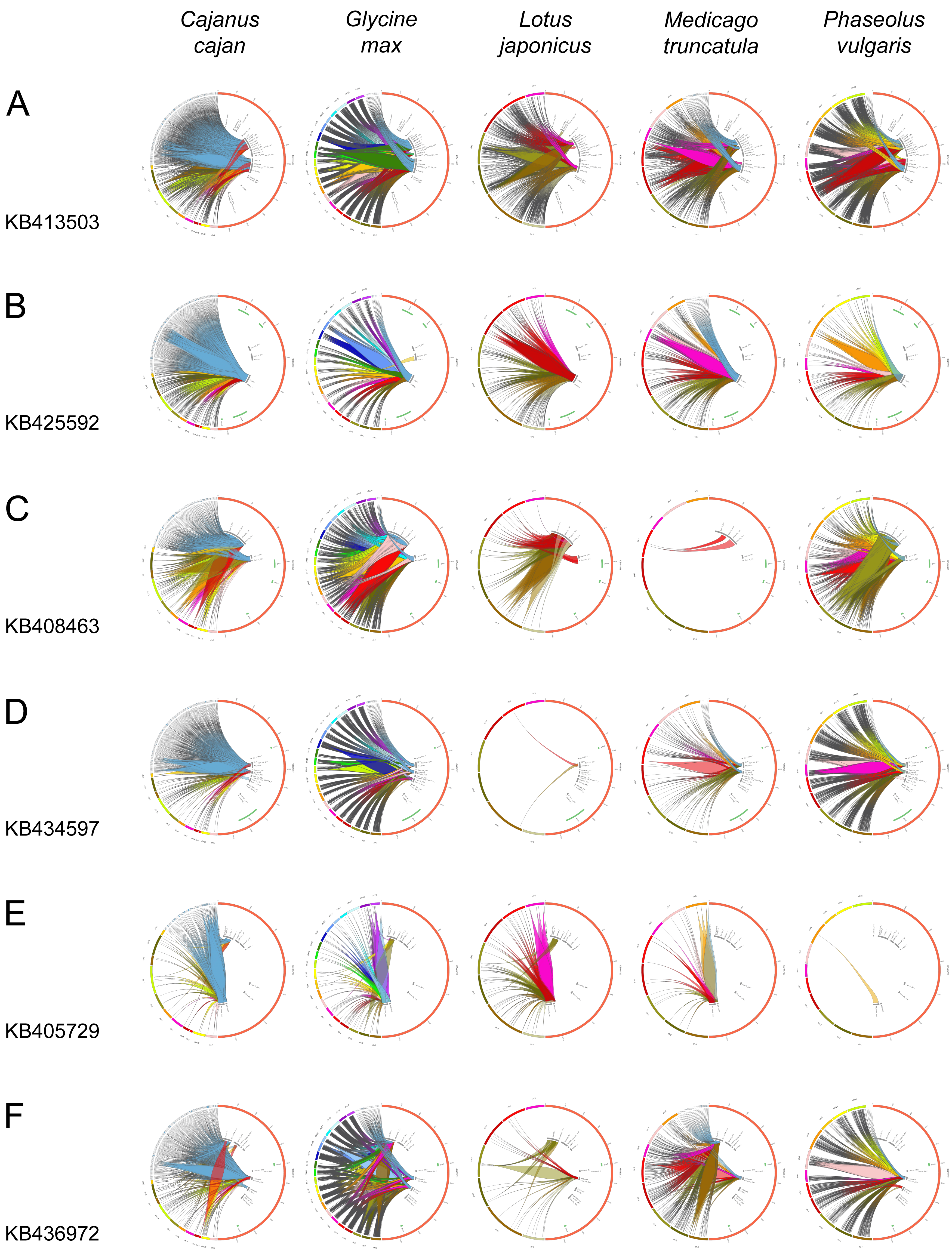

Cajanus cajan
Glycine max
Lotus japonicus
Medicago truncatula
Phaseolus vulgaris
A
KB413503
B
KB425592
C
KB408463
D
KB434597
E
KB405729
F
KB436972

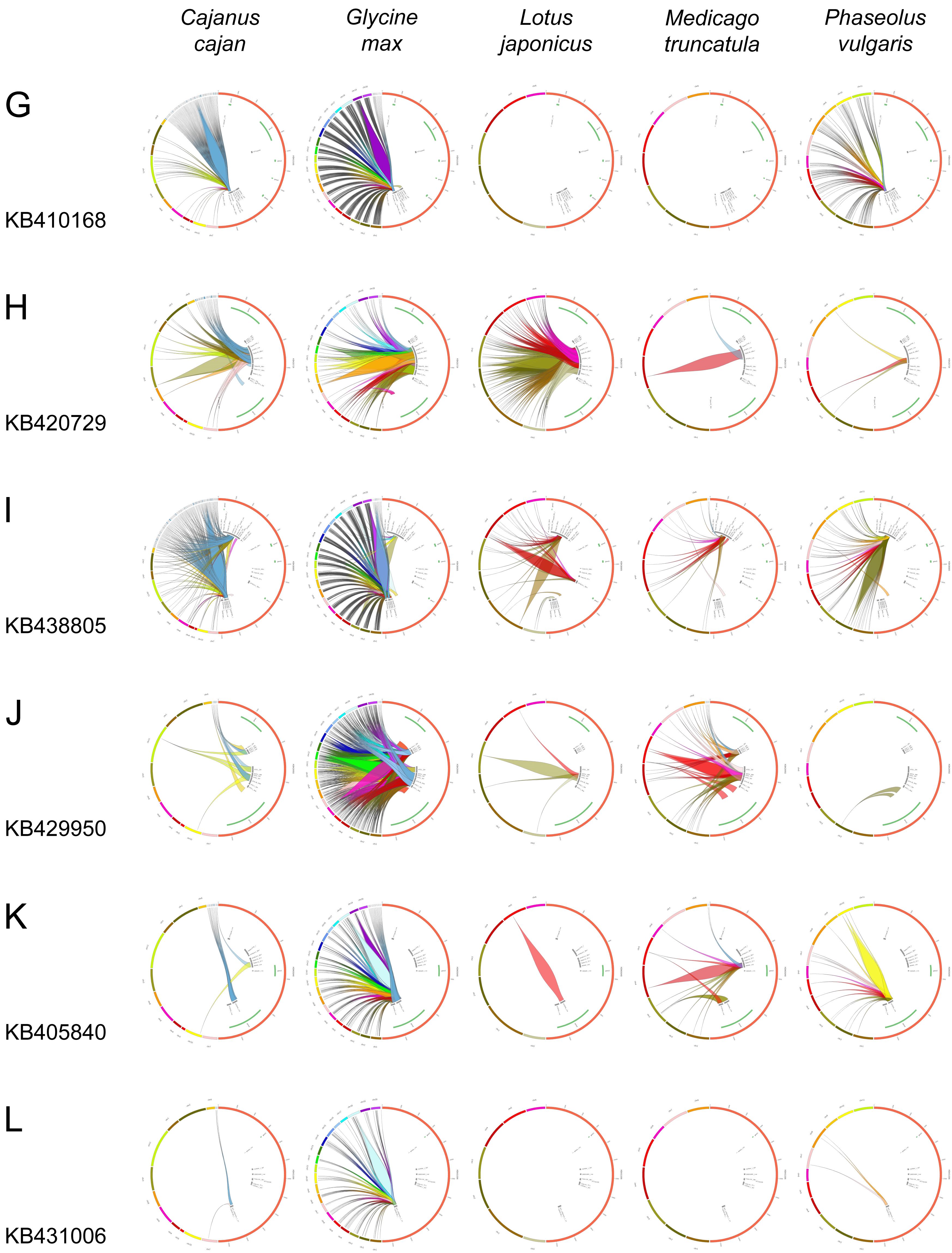

Cajanus cajan
Glycine max
Lotus japonicus
Medicago truncatula
Phaseolus vulgaris
G
KB410168
H
KB420729
I
KB438805
J
KB429950
K
KB405840
L
KB431006
