# Supplementary material for: Remnants of the Legume Ancestral Genome Preserved in Gene-Rich Regions: Insights from Lupinus angustifolius Physical, Genetic, and Comparative Mapping
Source: Plant Mol Biol Report. 2014 May 15;33(1):84–101. doi: 10.1007/s11105-014-0730-4 (PMC4295026; doi:10.1007/s11105-014-0730-4)
Supplement: Supplementary file 5 — Summary of scaffold annotation (PDF 18 kb) [file 11105_2014_730_MOESM5_ESM.pdf]

Summary of scaffold annotation

BES name	GI	Accession	BES length (nt)	Scaffold length (nt)	Sequence identity (%)	BES coverage (%)	Alignment e-value	Non-repetitive gene coverage (nt)	Non-repetitive gene coverage (%)	SSR length (nt)	SSR length (%)	Other repeat length (nt)	Other repeat length (%)	No. of non-repetitive genes	Mean gene density (genes / 100 kb)
096M15_5	451873247	KB425592.1	435	12563	99.54	100.00	0.00e+00	2427	19.32	1194	9.50	2386	18.99	4	31.84
024F15_5	451869649	KB429190.1	560	18431	99.64	99.82	0.00e+00	2627	14.25	1690	9.17	1241	6.73	5	27.13
080M14_5	451890376	KB408463.1	187	12409	100.00	100.00	3.00e-94	909	7.33	1290	10.40	3419	27.55	3	24.18
044I16_5	451888671	KB410168.1	215	21882	99.53	100.00	5.00e-108	3697	16.9	1988	9.09	2331	10.65	5	22.85
065A19_3	451887015	KB411824.1	833	19049	99.28	99.64	0.00e+00	2979	15.64	1799	9.44	3628	19.05	4	21.00
072B21_3	451890073	KB408766.1	751	14425	99.59	97.07	0.00e+00	657	4.55	1083	7.51	2907	20.15	3	20.80
026D07_5	451874300	KB424539.1	371	10382	100.00	100.00	0.00e+00	369	3.55	1159	11.16	3699	35.63	2	19.26
119M19_3	451868889	KB429950.1	477	10677	98.74	100.00	0.00e+00	4214	39.47	1302	12.19	2470	23.13	2	18.73
015P08_3	451865204	KB433635.1	615	26894	99.84	99.84	0.00e+00	5334	19.83	2558	9.51	3134	11.65	5	18.59
024B20_5	451892999	KB405840.1	164	11233	100.00	100.00	2.00e-81	2990	26.62	1025	9.12	3090	27.51	2	17.80
016M01_5	451890748	KB408091.1	559	50567	99.10	99.64	0.00e+00	9829	19.44	5621	11.12	7591	15.01	9	17.80
075D16_3	451861114	KB437725.1	588	11701	99.83	99.83	0.00e+00	2911	24.88	1139	9.73	847	7.24	2	17.09
015P23_5	451892290	KB406549.1	642	23709	99.84	100.00	0.00e+00	5936	25.04	2521	10.63	1218	5.14	4	16.87
080K03_3	451878260	KB420579.1	791	23794	99.37	100.00	0.00e+00	1839	7.73	1964	8.25	6589	27.69	4	16.81
072A20_3	451860034	KB438805.1	803	24397	99.63	99.75	0.00e+00	988	4.05	2302	9.44	6804	27.89	4	16.40
072A21_5	451882086	KB416753.1	618	18416	99.84	99.68	0.00e+00	3966	21.54	1301	7.06	2883	15.65	3	16.29
107M16_3	451864866	KB433973.1	712	74051	99.29	99.02	0.00e+00	30044	40.57	7987	10.79	54	0.07	12	16.21
140K16_3	451873888	KB424951.1	794	51666	99.50	100.00	0.00e+00	5529	10.7	4540	8.79	11031	21.35	8	15.48
060B20_5	451868498	KB430341.1	755	13444	100.00	99.60	0.00e+00	438	3.26	1392	10.35	2903	21.59	2	14.88
075D16_5	451875659	KB423180.1	543	20175	98.71	99.82	0.00e+00	5350	26.52	2367	11.73	0	0.00	3	14.87
115C21_3	451864176	KB434663.1	801	20747	99.25	99.50	0.00e+00	8132	39.2	2202	10.61	4893	23.58	3	14.46
024F17_3	451886110	KB412729.1	586	20791	99.83	100.00	0.00e+00	7101	34.15	2259	10.87	980	4.71	3	14.43
109D22_3	451857742	KB441097.1	832	14660	99.76	100.00	0.00e+00	435	2.97	1268	8.65	6709	45.76	2	13.64
015G12_5	451878500	KB420339.1	255	22040	99.61	100.00	1.00e-130	5020	22.78	2420	10.98	1321	5.99	3	13.61
112N18_5	451878110	KB420729.1	686	15364	98.54	99.27	0.00e+00	5729	37.29	1415	9.21	4876	31.74	2	13.02
064J10_3	451876834	KB422005.1	766	23187	99.73	96.08	0.00e+00	528	2.28	2536	10.94	4692	20.24	3	12.94
131L24_3	451883472	KB415367.1	708	39285	99.57	99.01	0.00e+00	4391	11.18	4403	11.21	8061	20.52	5	12.73
136B16_5	451881282	KB417557.1	535	17026	99.44	99.81	0.00e+00	5428	31.88	1979	11.62	331	1.94	2	11.75
024B13_3	451874529	KB424310.1	596	18378	99.83	100.00	0.00e+00	1952	10.62	1919	10.44	1760	9.58	2	10.88
017B07_3	451865892	KB432947.1	456	47059	99.78	100.00	0.00e+00	37682	80.07	5308	11.28	304	0.65	5	10.62
025M16_3	451892986	KB405853.1	368	10159	99.18	100.00	0.00e+00	1368	13.47	1191	11.72	0	0.00	1	9.84
120E23_5	451865298	KB433541.1	671	10528	98.46	95.68	0.00e+00	1523	14.47	673	6.39	666	6.33	1	9.50
051P10_3	451887709	KB411130.1	719	10638	100.00	100.00	0.00e+00	3978	37.39	1239	11.65	382	3.59	1	9.40
037D21_3	451882968	KB415871.1	739	32655	100.00	100.00	0.00e+00	12855	39.37	3462	10.60	3806	11.66	3	9.19
110J23_3	451892018	KB406821.1	804	21846	99.50	99.63	0.00e+00	1859	8.51	1900	8.70	7916	36.24	2	9.15
042C18_5	451861867	KB436972.1	704	21913	99.71	96.02	0.00e+00	663	3.03	1502	6.85	7454	34.02	2	9.13
128I22_5	451882501	KB416338.1	960	34573	99.90	100.00	0.00e+00	846	2.45	2824	8.17	9332	26.99	3	8.68
123A20_5	451861643	KB437196.1	1376	11638	98.69	99.71	0.00e+00	682	5.86	704	6.05	0	0.00	1	8.59
140A12_5	451864242	KB434597.1	640	23674	99.53	100.00	0.00e+00	3037	12.83	2169	9.16	4313	18.22	2	8.45
015L10_3	451872379	KB426460.1	593	36701	99.66	99.83	0.00e+00	12814	34.91	3806	10.37	9418	25.66	3	8.17
096O20_3	451890493	KB408346.1	550	24701	98.36	99.09	0.00e+00	4457	18.04	2765	11.19	969	3.92	2	8.10
122H24_3	451876056	KB422783.1	629	24780	98.35	96.18	0.00e+00	9920	40.03	2736	11.04	0	0.00	2	8.07
024F15_3	451888495	KB410344.1	683	25823	99.56	100.00	0.00e+00	1436	5.56	2823	10.93	9350	36.21	2	7.75
048P24_5	451868874	KB429965.1	505	41406	98.42	100.00	0.00e+00	2079	5.02	3561	8.60	10639	25.69	3	7.25
064B20_5	451882998	KB415841.1	746	43405	99.33	100.00	0.00e+00	1766	4.07	3875	8.93	12510	28.82	3	6.91
084P14_5	451893577	KB405262.1	922	17506	99.78	99.89	0.00e+00	459	2.62	1464	8.36	6317	36.08	1	5.71
043B20_5	451873586	KB425253.1	668	17566	99.40	100.00	0.00e+00	333	1.9	2010	11.44	5632	32.06	1	5.69
100A19_5	451867833	KB431006.1	781	23124	99.61	98.72	0.00e+00	102	0.44	1834	7.93	2333	10.09	1	4.32
080F20_5	451880266	KB418573.1	787	29847	99.24	99.62	0.00e+00	210	0.7	3024	10.13	10197	34.16	1	3.35
072A20_5	451879746	KB419093.1	746	33814	99.87	100.00	0.00e+00	48	0.14	2911	8.61	8943	26.45	1	2.96
112E01_3	451888260	KB410579.1	759	34309	99.47	99.87	0.00e+00	1844	5.37	2478	7.22	11091	32.33	1	2.91
064B20_3	451867183	KB431656.1	785	13548	99.87	100.00	0.00e+00	0	0	1089	8.04	7273	53.68	0	0.00
064H23_3	451872828	KB426011.1	792	28117	99.75	100.00	0.00e+00	0	0	2624	9.33	12022	42.76	0	0.00
103A04_5	451874893	KB423946.1	570	11680	99.12	99.65	0.00e+00	0	0	1336	11.44	4796	41.06	0	0.00
072A21_3	451867578	KB431261.1	815	22105	99.88	98.90	0.00e+00	0	0	2298	10.40	9063	41.00	0	0.00
109D22_5	451885336	KB413503.1	184	23190	100.00	98.91	2.00e-91	0	0	2342	10.10	8825	38.06	0	0.00
072B21_5	451893110	KB405729.1	680	13061	99.85	99.56	0.00e+00	0	0	695	5.32	4347	33.28	0	0.00
