# Supplementary material for: Remnants of the Legume Ancestral Genome Preserved in Gene-Rich Regions: Insights from Lupinus angustifolius Physical, Genetic, and Comparative Mapping
Source: Plant Mol Biol Report. 2014 May 15;33(1):84–101. doi: 10.1007/s11105-014-0730-4 (PMC4295026; doi:10.1007/s11105-014-0730-4)
Supplement: Supplementary file 6 — Linkage groups of the L. angustifolius genetic map supplemented with new genetic markers (PDF 623 kb) [file 11105_2014_730_MOESM6_ESM.pdf]

Linkage groups of the *L. angustifolius* genetic map (Nelson et al. 2010, Książkiewicz et al. 2013) supplemented with new genetic markers

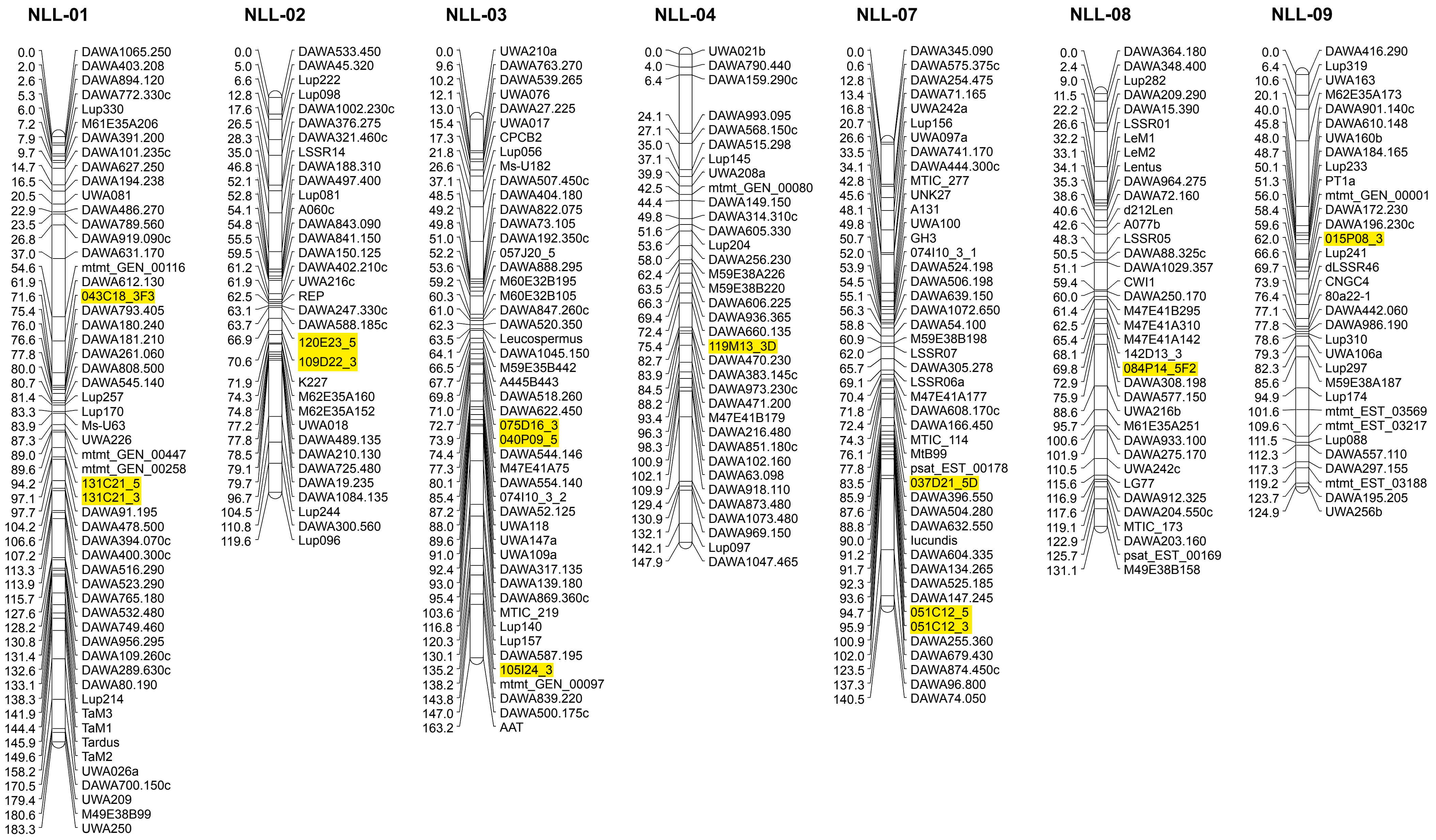

New markers are highlighted in yellow. Map distances are given in Kosambi centimorgans.

References:

Nelson MN, Moolhuijzen PM, Boersma JG, et al. (2010) Aligning a new reference genetic map of Lupinus angustifolius with the genome sequence of the model legume, Lotus japonicus. DNA Res Int J Rapid Publ Rep Genes Genomes 17:73–83

Książkiewicz M, Wyrwa K, Szczepaniak A, et al. (2013) Comparative genomics of Lupinus angustifolius gene-rich regions: BAC library exploration, genetic mapping and cytogenetics. BMC Genomics 14:79

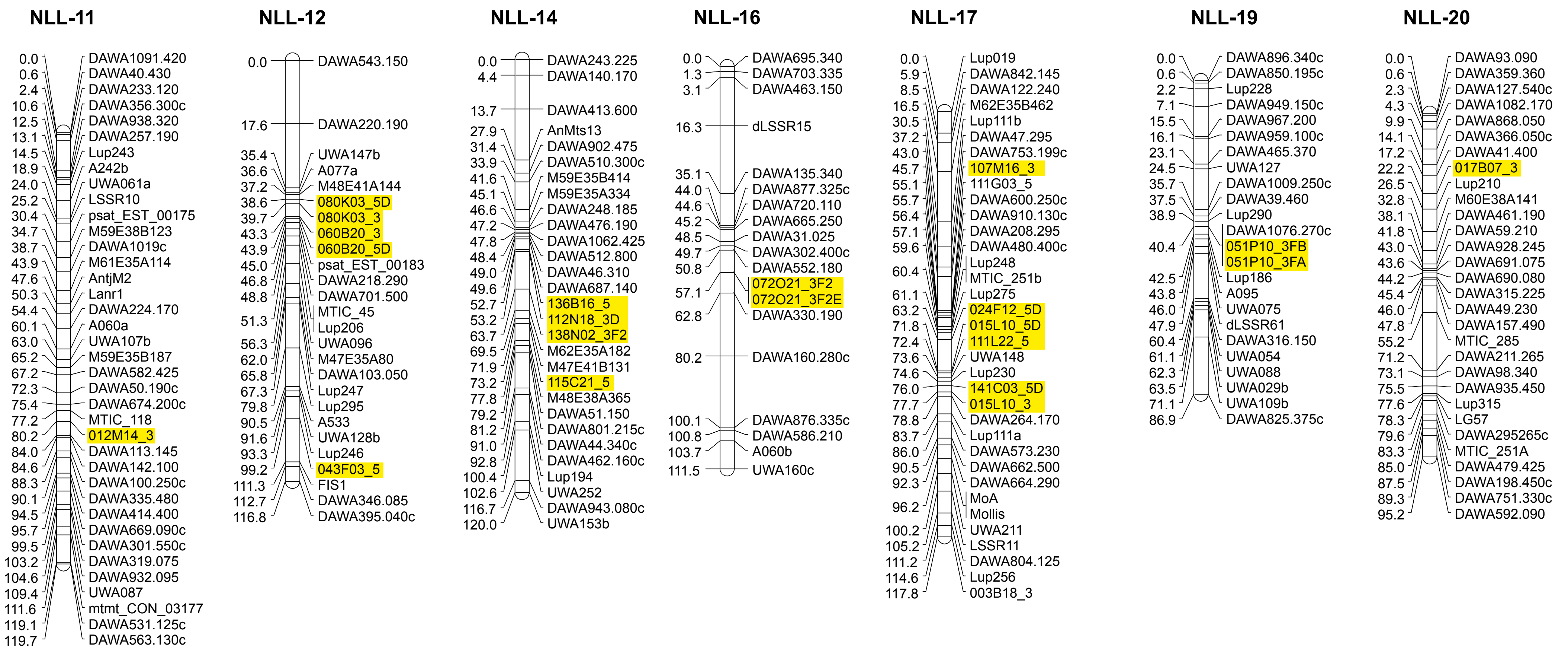
NLL-11
0.0 DAWA1091.420
0.6 DAWA40.430
2.4 DAWA233.120
10.6 DAWA356.300c
12.5 DAWA938.320
13.1 DAWA257.190
14.5 Lup243
18.9 A242b
24.0 UWA061a
25.2 LSSR10
30.4 psat_EST_00175
34.7 M59E38B123
38.7 DAWA1019c
43.9 M61E35A114
47.6 AntjM2
50.3 Lanr1
54.4 DAWA224.170
60.1 A060a
63.0 UWA107b
65.2 M59E35B187
67.2 DAWA582.425
72.3 DAWA50.190c
75.4 DAWA674.200c
77.2 MTIC_118
80.2 012M14_3
84.0 DAWA113.145
84.6 DAWA142.100
88.3 DAWA100.250c
90.1 DAWA335.480
94.5 DAWA414.400
95.7 DAWA669.090c
99.5 DAWA301.550c
103.2 DAWA319.075
104.6 DAWA932.095
109.4 UWA087
111.6 mtmt_CON_03177
119.1 DAWA531.125c
119.7 DAWA563.130c
NLL-12
0.0 DAWA543.150
17.6 DAWA220.190
35.4 UWA147b
36.6 A077a
37.2 M48E41A144
38.6 080K03_5D
39.7 080K03_3
43.3 060B20_3
43.9 060B20_5D
45.0 psat_EST_00183
46.8 DAWA218.290
48.8 DAWA701.500
51.3 MTIC_45
Lup206
56.3 UWA096
62.0 M47E35A80
65.8 DAWA103.050
67.3 Lup247
79.8 Lup295
90.5 A533
91.6 UWA128b
93.3 Lup246
99.2 043F03_5
111.3 FIS1
112.7 DAWA346.085
116.8 DAWA395.040c
NLL-14
0.0 DAWA243.225
4.4 DAWA140.170
13.7 DAWA413.600
27.9 AnMts13
31.4 DAWA902.475
33.9 DAWA510.300c
41.6 M59E35B414
45.1 M59E35A334
46.6 DAWA248.185
47.2 DAWA476.190
47.8 DAWA1062.425
48.4 DAWA512.800
49.0 DAWA46.310
49.6 DAWA687.140
52.7 136B16_5
53.2 112N18_3D
63.7 138N02_3F2
69.5 M62E35A182
71.9 M47E41B131
73.2 115C21_5
77.8 M48E38A365
79.2 DAWA51.150
81.2 DAWA801.215c
91.0 DAWA44.340c
92.8 DAWA462.160c
100.4 Lup194
102.6 UWA252
116.7 DAWA943.080c
120.0 UWA153b
NLL-16
0.0 DAWA695.340
1.3 DAWA703.335
3.1 DAWA463.150
16.3 dLSSR15
35.1 DAWA135.340
44.0 DAWA877.325c
44.6 DAWA720.110
45.2 DAWA665.250
48.5 DAWA31.025
49.7 DAWA302.400c
50.8 DAWA552.180
57.1 072O21_3F2
072O21_3F2E
62.8 DAWA330.190
80.2 DAWA160.280c
100.1 DAWA876.335c
100.8 DAWA586.210
103.7 A060b
111.5 UWA160c
NLL-17
0.0 Lup019
5.9 DAWA842.145
8.5 DAWA122.240
16.5 M62E35B462
30.5 Lup111b
37.2 DAWA47.295
43.0 DAWA753.199c
45.7 107M16_3
55.1 111G03_5
55.7 DAWA600.250c
56.4 DAWA910.130c
57.1 DAWA208.295
59.6 DAWA480.400c
60.4 Lup248
MTIC_251b
61.1 Lup275
63.2 024F12_5D
71.8 015L10_5D
72.4 111L22_5
73.6 UWA148
74.6 Lup230
76.0 141C03_5D
77.7 015L10_3
78.8 DAWA264.170
83.7 Lup111a
86.0 DAWA573.230
90.5 DAWA662.500
92.3 DAWA664.290
96.2 MoA
Mollis
100.2 UWA211
105.2 LSSR11
111.2 DAWA804.125
114.6 Lup256
117.8 003B18_3
NLL-19
0.0 DAWA896.340c
0.6 DAWA850.195c
2.2 Lup228
7.1 DAWA949.150c
15.5 DAWA967.200
16.1 DAWA959.100c
23.1 DAWA465.370
24.5 UWA127
35.7 DAWA1009.250c
37.5 DAWA39.460
38.9 Lup290
40.4 DAWA1076.270c
051P10_3FB
051P10_3FA
42.5 Lup186
43.8 A095
46.0 UWA075
47.9 dLSSR61
60.4 DAWA316.150
61.1 UWA054
62.3 UWA088
63.5 UWA029b
71.1 UWA109b
86.9 DAWA825.375c
NLL-20
0.0 DAWA93.090
0.6 DAWA359.360
2.3 DAWA127.540c
4.3 DAWA1082.170
9.9 DAWA868.050
14.1 DAWA366.050c
17.2 DAWA41.400
22.2 017B07_3
26.5 Lup210
32.8 M60E38A141
38.1 DAWA461.190
41.8 DAWA59.210
43.0 DAWA928.245
43.6 DAWA691.075
44.2 DAWA690.080
45.4 DAWA315.225
46.0 DAWA49.230
47.8 DAWA157.490
55.2 MTIC_285
71.2 DAWA211.265
73.1 DAWA98.340
75.5 DAWA935.450
77.6 Lup315
78.3 LG57
79.6 DAWA295265c
83.3 MTIC_251A
85.0 DAWA479.425
87.5 DAWA198.450c
89.3 DAWA751.330c
95.2 DAWA592.090
