# Supplementary material for: Remnants of the Legume Ancestral Genome Preserved in Gene-Rich Regions: Insights from Lupinus angustifolius Physical, Genetic, and Comparative Mapping
Source: Plant Mol Biol Report. 2014 May 15;33(1):84–101. doi: 10.1007/s11105-014-0730-4 (PMC4295026; doi:10.1007/s11105-014-0730-4)
Supplement: Supplementary file 1 — Functional annotation of BAC-end sequences (PDF 90 kb) [file 11105_2014_730_MOESM1_ESM.pdf]

Functional annotation of BAC-end sequences

BAC	Contig	BAC-FISH signals	BAC-end sequence (BES) name	Accession	RepeatMasker - repeat 1 BES start	RepeatMasker - repeat 1 BES end	RepeatMasker - repeat 1 Score	RepeatMasker - repeat 1 Repeat name	RepeatMasker - repeat 1 Repeat type	RepeatMasker - repeat 2 BES start	RepeatMasker - repeat 2 BES end	RepeatMasker - repeat 2 Score	RepeatMasker - repeat 2 Repeat name	RepeatMasker - repeat 2 Repeat type	RepeatMasker - repeat 3 BES start	RepeatMasker - repeat 3 BES end	RepeatMasker - repeat 3 Score	RepeatMasker - repeat 3 Repeat name	RepeatMasker - repeat 3 Repeat type	Repbase blastn BES start	Repbase blastn BES end	Repbase blastn Max score	Repbase tblastx BES start	Repbase tblastx BES end	Repbase tblastx Max score	Repeat summary BES start	Repeat summary BES end	Repeat summary Name	*Lupinus albus* EST Subject ID	*Lupinus albus* EST BES start	*Lupinus albus* EST BES end	*Lupinus albus* EST E-value	*Lupinus luteus* EST Subject ID	*Lupinus luteus* EST BES start	*Lupinus luteus* EST BES end	*Lupinus luteus* EST E-value	Fabaceae EST Subject ID	Fabaceae EST BES start	Fabaceae EST BES end	Fabaceae EST E-value	*Glycine* EST Subject ID	*Glycine* EST BES start	*Glycine* EST BES end	*Glycine* EST E-value	*Lotus* EST Subject ID	*Lotus* EST BES start	*Lotus* EST BES end	*Lotus* EST E-value	*Lupinus* EST Subject ID	*Lupinus* EST BES start	*Lupinus* EST BES end	*Lupinus* EST E-value	*Medicago* EST Subject ID	*Medicago* EST BES start	*Medicago* EST BES end	*Medicago* EST E-value	*Phaseolus* EST Subject ID	*Phaseolus* EST BES start	*Phaseolus* EST BES end	*Phaseolus* EST E-value	Viridiplaneae SwissProt Subject ID	Viridiplaneae SwissProt name	Viridiplaneae SwissProt BES start	Viridiplaneae SwissProt BES end	Viridiplaneae SwissProt E-value	Viridiplaneae trEMBL Subject ID	Viridiplaneae trEMBL Protein name	Viridiplaneae trEMBL BES start	Viridiplaneae trEMBL BES end	Viridiplaneae trEMBL E-value
008A03	9	single locus	008A03_3	AB809166	4	577	4310	SSU-rRNA_Ath	rRNA	-	-	-	-	-	-	-	-	-	-	4	570	738	1	578	345	1	578	rRNA	I2itg00057	4	597	0	LAGI01_66161	4	594	0	gi|241807930|gb|GR407169.1|GR407169	4	634	0	gi|14205340|gb|BG839018.1|BG839018	20	582	0	gi|93640387|gb|BW594631.1|BW594631	8	486	0	gi|27460146|gb|CA411142.1|CA411142	4	594	0	gi|89245594|gb|DY617381.1|DY617381	4	630	0	gi|308768687|gb|HO774974.1|HO774974	4	594	0	-	-	-	-	-	tr|G7K0D6|G7K0D6_MEDTR	RRNA intron-encoded homing endonuclease	313	510	2e-26
			008A03_5	AB809167	1364	1490	226	ATCopia76_I	LTR/Copia	-	-	-	-	-	-	-	-	-	-	1	2211	2091	1	2212	266	1	2212	LTR/Copia	I0itg05047	260	1623	0	LAGI01_81601	1773	2166	3e-149	gi|29969344|gb|CB828938.1|CB828938	1	245	4e-82	gi|15000988|gb|BI321802.1|BI321802	508	862	9e-42	gi|29969344|gb|CB828938.1|CB828938	1	245	4e-83	-	-	-	-	-	-	-	-	-	-	-	-	sp|P10978|POLX_TOBAC	Retrovirus-related Pol polyprotein from transposon TNT 1-94	1056	1463	4e-31	tr|A5BE46|A5BE46_VITVI	Hypothetical protein	1	984	4e-157
008K02	5	n	008K02_3	AB809168	335	485	313	(CAA)n	Simple_repeat	-	-	-	-	-	-	-	-	-	-	-	-	-	-	-	-	-	-	-	I0itg56254	102	364	6e-74	LAGI01_64750	1	91	2e-19	gi|86122800|gb|BB934009.1|BB934009	340	482	2e-11	-	-	-	-	-	-	-	-	-	-	-	-	-	-	-	-	-	-	-	-	-	-	-	-	-	-	-	-	-	-
			008K02_5	AB809169	4	274	375	ATHAT8	DNA/hAT-Ac	-	-	-	-	-	-	-	-	-	-	2	505	72,5	2	601	113	2	601	DNA/hAT	I2itg21521	1	104	5e-37	LAGI01_64750	2	606	0	gi|148764212|gb|ES610906.1|ES610906	1	200	3e-40	-	-	-	-	gi|45574882|gb|BP042244.1|BP042244	3	121	1e-19	-	-	-	-	gi|148764212|gb|ES610906.1|ES610906	1	200	3e-41	-	-	-	-	sp|P08770|TRA1_MAIZE	Putative AC transposase	589	2	3e-29	tr|G7K7Q6|G7K7Q6_MEDTR	Oligoribonuclease	604	20	9e-61
012M14	0	repetitive	012M14_3	HR864171	-	-	-	-	-	-	-	-	-	-	-	-	-	-	-	-	-	-	-	-	-	-	-	-	I2itg05551	3	143	3e-43	-	-	-	-	-	-	-	-	-	-	-	-	-	-	-	-	-	-	-	-	-	-	-	-	-	-	-	-	-	-	-	-	-	-	-	-	-	-
			012M14_5	AB809170	1	143	381	(TGGGGG)n	Simple_repeat	158	322	281	(GGA)n	Simple_repeat	319	517	307	(GGA)n	Simple_repeat	-	-	-	-	-	-	-	-	-	I2itg13080	65	580	5e-68	-	-	-	-	-	-	-	-	-	-	-	-	-	-	-	-	-	-	-	-	-	-	-	-	-	-	-	-	-	-	-	-	-	-	-	-	-	-
015G12	5	n	015G12_3	AB809171	366	391	26	AT_rich	Low_complexity	-	-	-	-	-	-	-	-	-	-	-	-	-	-	-	-	-	-	-	I0ctg64560	23	533	0	-	-	-	-	-	-	-	-	-	-	-	-	-	-	-	-	-	-	-	-	-	-	-	-	-	-	-	-	-	-	-	-	-	-	-	-	-	-
			015G12_5	AB809172	-	-	-	-	-	-	-	-	-	-	-	-	-	-	-	-	-	-	5	208	51,5	5	208	LTR/Copia	-	-	-	-	-	-	-	-	-	-	-	-	-	-	-	-	-	-	-	-	-	-	-	-	-	-	-	-	-	-	-	-	-	-	-	-	-	-	-	-	-	-
015L10	3	repetitive	015L10_3	HR864172	-	-	-	-	-	-	-	-	-	-	-	-	-	-	-	-	-	-	-	-	-	-	-	-	I2itg31382	286	592	3e-70	LAGI01_62100	171	495	5e-51	gi|73845511|gb|DT454531.1|DT454531	1	75	5e-30	gi|6846476|gb|AW348766.1|AW348766	1	74	5e-19	gi|13883992|gb|BG662070.1|BG662070	1	74	1e-19	gi|73845511|gb|DT454531.1|DT454531	1	75	2e-32	gi|7675410|gb|AW328958.2|AW328958	1	74	1e-20	gi|27384851|gb|CA897860.1|CA897860	1	74	2e-19	-	-	-	-	-	-	-	-	-	-
			015L10_5	HR864204	-	-	-	-	-	-	-	-	-	-	-	-	-	-	-	-	-	-	-	-	-	-	-	-	I2itg04224	28	664	4e-70	-	-	-	-	-	-	-	-	-	-	-	-	-	-	-	-	-	-	-	-	-	-	-	-	-	-	-	-	-	-	-	-	-	-	-	-	-	-
015P08	1	single locus	015P08_3	AB809173	-	-	-	-	-	-	-	-	-	-	-	-	-	-	-	-	-	-	-	-	-	-	-	-	-	-	-	-	-	-	-	-	-	-	-	-	-	-	-	-	-	-	-	-	-	-	-	-	-	-	-	-	-	-	-	-	-	-	-	-	-	-	-	-	-	-	-
			015P08_5	AB809174	5	535	589	ATCopia38B_I	LTR/Copia	-	-	-	-	-	-	-	-	-	-	3	551	149	1	582	270	1	582	LTR/Copia	I2itg08197	7	553	5e-69	LAGI01_55670	7	546	1e-40	gi|223447239|gb|GO039125.1|GO039125	188	548	3e-34	-	-	-	-	gi|223447239|gb|GO039125.1|GO039125	188	548	3e-35	-	-	-	-	gi|148765576|gb|ES612270.1|ES612270	7	350	1e-14	gi|312033252|gb|GW885225.1|GW885225	196	516	1e-14	sp|P92519|M810_ARATH	Uncharacterized mitochondrial protein AtMg00810	4	372	2e-28	tr|Q2HU08|Q2HU08_MEDTR	Polyprotein-like	1	597	1e-68
[illegible]	[illegible]	[illegible]	[illegible]	[illegible]	[illegible]	[illegible]	[illegible]	[illegible]	[illegible]	[illegible]	[illegible]	[illegible]	[illegible]	[illegible]	[illegible]	[illegible]	[illegible]	[illegible]	[illegible]	[illegible]	[illegible]	[illegible]	[illegible]	[illegible]	[illegible]	[illegible]	[illegible]	[illegible]	[illegible]	[illegible]	[illegible]	[illegible]	[illegible]	[illegible]	[illegible]	[illegible]	[illegible]	[illegible]	[illegible]	[illegible]	[illegible]	[illegible]	[illegible]	[illegible]	[illegible]	[illegible]	[illegible]	[illegible]	[illegible]	[illegible]	[illegible]	[illegible]	[illegible]	[illegible]	[illegible]	[illegible]	[illegible]	[illegible]	[illegible]	[illegible]	[illegible]	[illegible]	[illegible]	[illegible]	[illegible]	[illegible]	[illegible]	[illegible]	[illegible]	[illegible]

n - clone not analyzed in BAC-FISH
x - low quality sequence
\- - no significant alignment
