# Supplementary material for: Remnants of the Legume Ancestral Genome Preserved in Gene-Rich Regions: Insights from Lupinus angustifolius Physical, Genetic, and Comparative Mapping
Source: Plant Mol Biol Report. 2014 May 15;33(1):84–101. doi: 10.1007/s11105-014-0730-4 (PMC4295026; doi:10.1007/s11105-014-0730-4)
Supplement: Supplementary file 7 — Data on the genetic markers developed in this study (PDF 53 kb) [file 11105_2014_730_MOESM7_ESM.pdf]

Data on the genetic markers developed in this study

Marker	Contig	Accessions 83A:476	Accessions P27255	Primer pair sequences (dCAPS primers underlined)	Annealing temperature	PCR product length for BAC DNA	PCR product length for 83A:476 DNA	PCR product length for P27255 DNA	BAC/83A:476 sequence identity	83A:476/P27255 sequence identity	Polymorphism detection	83A:476 product(s) length	P27255 product(s) length	NLL linkage group	LOD scores		Segregation data ("-" no polymorphism, "0" no data, "1" genotype 83A:476, "2" genotype P27255, "H" heterozygote) W/D 8	W/D 9	W/D 10	W/D 11–136, 83A:476, P27255
008A03_5	9	AB811081	AB811255	F:AGGTGTCGGAAGACTTACTCAT R:AAATGCCATATAGCTTCCACTT	63	550	550	550	535/550 (98%)	550/550 (100%)	-	-	-	-	-	-	-	-	-	-
008A03_5F2	9	AB811082	AB811256	F:TGGTTAACTGGTTTCTATTT R:CGTCTTGAAGCTTCTTATATGCAACC	46	456	452	452	445/456(98%)	452/452(100%)	-	-	-	-	-	-	-	-	-	-
008A03_5F3	9	AB811083	AB811257	F:GGTTGCATATAAGAAGCTTCAAGACG R:GCTCATCAAGGTGTTTTAGCAG	60	527	525	525	521/527(99%)	525/525(100%)	-	-	-	-	-	-	-	-	-	-
012M14_3	0	AB811084	-	F:TGTGTCCTAACATTTCCTCTTTC R:ATACTCTTCCTCATGCAATCCT	58	300	300	-	298/302 (99%)	-	AS-PCR	300	-	NLL-11	18.2	16.2	2	1	1	[illegible]
015L10_3	3	AB811085	AB811258	F:CTTGTTTACCAGACCACTAAGAA R:TACACATTCATCAATGAGAAAAA	46	440	440	440	440/440(100%)	431/442 (97%)	CAPS, ***Tas*I**	13, 25, 36, 95, 110, 161	13, 25, 28, 36, 84, 95, 159	NLL-12	21.4	22.3	2	1	2	[illegible]
015L10_5	3	AB811086	AB811259	F:TTGTTCTTTCGTTTGATTTATTT R:GCAACCATCTTAAGACTTCTAAT	46	404	404	404	404/404 (100%)	403/404 (99%)	-	-	-	-	-	-	-	-	-	-
015L10_5D	3	AB811087	AB811260	F:<u>GGAAGCAACACAACTTTGATATATGGC</u> R:GCAACCATCTTAAGACTTCTAAT	58	293	293	293	291/293(99%)	292/293(99%)	dCAPS, ***Bsu*RI**	27, 266	293	NLL-17	10.7	24.4	2	1	2	[illegible]
015P08_3	1	AB811088	-	F:GCGCCTTCATCTTCCTATTTA R:TTCAAAGGCTTACACCCGAATA	56	551	550	-	550/551(99%)	-	AS-PCR	550	-	NLL-09	18.9	12.7	2	2	2	[illegible]
015P22_3	9	AB811089	AB811261	F:AAGCTTATTATCTCAAGTGGACA R:CAGAGGAGAACTTTCTATTCAAC	48	503	502	502	479/504 (95%)	502/502 (100%)	-	-	-	-	-	-	-	-	-	-
015P22_5	9	AB811090	AB811262	F:TCCTCATTAGAACTGGATAAAAG R:ATGAATGTAGGAGTAGAGTTCCA	48	435	435	435	421/435 (97%)	435/435 (100%)	-	-	-	-	-	-	-	-	-	-
015P23_3	0	AB811091	AB811263	F:CTTTACACGGCCAGACTC R:TTTGTATCTCATTTTCCATCTC	48	588	561	561	227/298 (76%)	551/561(98%)	-	-	-	-	-	-	-	-	-	-
017B07_3	1	AB811092	AB811264	F:TTTACTTAAGAGGCTTTTCCTC R:TCACTACGTGCTTCGTATTC	51	318	317	317	316/318 (99%)	316/317 (99%)	CAPS, ***Taq*I**	317	141, 176	NLL-20	14.8	13.8	2	2	2	[illegible]
017B07_5	1	AB811093	AB811265	F:ATTGCGGAGCAGGTTATTTAT R:TATTACACTCCCTCACCCCAGT	54	403	402	402	401/403 (99%)	402/402 (100%)	-	-	-	-	-	-	-	-	-	-
024B20_3	11	AB811094	AB811266	F:GCTTACATGTCTTTACATGCTT R:TGTGACAAGAATAGACACAAAA	48	462	405	405	286/369 (78%)	405/405 (100%)	-	-	-	-	-	-	-	-	-	-
024F12_3	11	AB811095	AB811267	F:GAGCTCCTTCTTGATTCTATTG R:TGCTTCCTCTAAATGATAAGGT	50	514	515	515	478/516 (92%)	515/515 (100%)	-	-	-	-	-	-	-	-	-	-
024F12_5	11	AB811096	AB811268	F:GCTTCTCTGGCTTGTGC R:AAGACATGATTTAGGATAGTAGAT	46	385	385	385	385/385 (100%)	382/385 (99%)	-	-	-	-	-	-	-	-	-	-
024F12_5D	11	AB811097	-	F:GCTTCTCTGGCTTGTGC R:GGATAGTAGATTATCCTTG	53	372	372	-	372/372(100%)	-	AS-PCR	372	-	NLL-17	16.5	10.7	1	1	1	[illegible]
030F13_3	7	AB811098	AB811269	F:CTTGTCCAAAAGCAGGTTCATA R:GCTGTTGAAATGTATAAGTCGTG	57	550	550	550	523/551 (95%)	550/550 (100%)	-	-	-	-	-	-	-	-	-	-
030F13_5	7	AB811099	AB811270	F:GATATGCTTGACATGATTTCG R:GAGAATAGGTGAGACAAAGAACA	60	287	287	287	285/287 (99%)	287/287 (100%)	-	-	-	-	-	-	-	-	-	-
037B11_3	0	AB811100	AB811271	F:TTGGTTTTGATGAGGACAACT R:TAGATGCAGCAATTAACACGA	63	572	567	567	551/572 (97%)	567/567 (100%)	-	-	-	-	-	-	-	-	-	-
037B11_5	0	AB811101	AB811272	F:GCTTTAGTGGTTTTGGGTATTG R:CGAGCCAAGAAACTGAAAATA	62	504	504	504	503/504 (99%)	504/504 (100%)	-	-	-	-	-	-	-	-	-	-
037D21_3	0	AB811102	AB811273	F:TGGAATACCATTATGAAAAATC R:CATATTAGCCATTTCCATCATA	52	618	617	617	617/618 (99%)	617/617 (100%)	-	-	-	-	-	-	-	-	-	-
037D21_5	0	AB811103	AB811274	F:GGTGCTCAGAAGTATCAATCT R:TCTTAACCATCAAACCATCTTA	56	452	451	451	450/452 (99%)	450/451 (99%)	-	-	-	-	-	-	-	-	-	-
037D21_5D	0	AB811104	AB811275	F:<u>ATATGCACTAAGTATCCAAAAAGCACA</u> R:TCTTAACCATCAAACCATCTTA	57	316	316	316	314/316(99%)	315/316(99%)	dCAPS, ***Hin*1II**	30, 82, 204	112, 204	NLL-07	14.1	19.4	2	2	1	[illegible]
040P09_3	0	AB811105	AB811276	F:CCAAATGTTCTATCTCTTTGC R:CTAAGACCAAAAAGGCCCAAG	62	358	358	358	356/358 (99%)	358/358 (100%)	-	-	-	-	-	-	-	-	-	-
040P09_5	0	AB811106	AB811277	F:GACCTTGCCTCCTTTCCTA R:TGAGGATAGAATGGGGATG	58	500	498	498	495/500 (99%)	497/498 (99%)	CAPS, ***Tas*I**	24, 39, 69, 73, 86, 207	24, 39, 73, 86, 276	NLL-03	22.9	23.8	1	1	1	[illegible]
043C18_3	1	AB811107	AB811278	F:AGAGCAGCCTTTAGGTAAGGAAG R:GGCCTTTAGCTCCTCCTTCTAC	58	350	360	360	346/363 (96%)	360/360 (100%)	-	-	-	-	-	-	-	-	-	-
043C18_3F2	1	AB811108	AB811279	F:AGTTTCGTAGTCAGCCAGGCAA R:GCGACGCACATAGTCTTAAGT	57	444	445	445	437/440(99%)	445/445(100%)	-	-	-	-	-	-	-	-	-	-
043C18_3F3	1	AB811109	-	F:GTCGCGTGCTCTATCTAGCAAT R:CGGTGAACTACACTTGCTTCTG	47	555	555	-	554/555(99%)	-	AS-PCR	555	-	NLL-01	9.5	16.2	2	2	1	[illegible]
043C18_5	1	AB811110	AB811280	F:GCCATGTAAACTTCTTTGCATCT R:AAGAGGATCTTCAGGAGTGTCC	68	461	455	455	439/461 (96%)	455/455 (100%)	-	-	-	-	-	-	-	-	-	-
043F03_3	13	AB811111	AB811281	F:CCTTGTTGAACCCTTAATTATC R:CCCTGAGTCTTCAACCTTTA	50	118	116	116	116/118 (98%)	116/116 (100%)	-	-	-	-	-	-	-	-	-	-
043F03_5	13	AB811112	AB811282	F:CCAATGTGCTTCTTCATATTAG R:AATATCTGATCCAGAATCCAAG	50	356	353	355	353/356 (99%)	353/355 (99%)	CAPS, ***Ssp*I**	92, 261	355	NLL-12	12.0	6.9	1	2	1	[illegible]
044J15_3	12	AB811113	AB811283	F:ATAACATTGTGGCTGACAATTA R:CTATGACATCCCTGTTGGAT	56	255	253	253	250/255 (98%)	253/253 (100%)	-	-	-	-	-	-	-	-	-	-
051C12_3	9	AB811114	-	F:AGTGATGTCAAAATGGATCG R:GAAATTCAGCATGTTAATCG	55	493	465	-	262/385 (68%)	-	AS-PCR	465	-	NLL-07	22.9	14.8	2	2	1	[illegible]
051C12_5	9	AB811115	AB811284	F:TAAAGGCAAGGACATATCAACA R:CAAAACTTGTCTTTCCTATACCC	55	501	499	499	499/501 (99%)	490/499 (99%)	CAPS, ***Tas*I**	43, 70, 77, 93, 216	42, 43, 51, 70, 77, 216	NLL-07	22.3	22.9	2	2	1	[illegible]
051P10_3	0	AB811116	AB811285	F:GGAAATTTTACTGCACGACTA R:CCTTTTGATGGTAAATGAAGC	57	603	601	612	601/603 (99%)	600/612 (99%)	-	-	-	-	-	-	-	-	-	-
051P10_3FA	0	AB811117	-	F:<u>TCCAAATGATCCAAATAAAA</u> R:CCTTTTGATGGTAAATGAAGC	44	212	212	-	212/212 (100%)	-	*AS-PCR*	212	-	NLL-19	22.6	26.5	1	1	1	[illegible]
051P10_3FB	0	-	AB811286	F:<u>TCCAAATGATCCATATATGATC</u> R:CCTTTTGATGGTAAATGAAGC	44	212	-	223	-	207/207(100%)	*AS-PCR*	-	223	NLL-19	26.5	17.1	1	1	1	[illegible]
051P10_5	0	-	-	F:CTTGGTAAGGCAAGTTTCTGAT R:CCTGAAAGAGATGCAAGTGTAA	60	551	sequence unclear	sequence unclear	-	-	-	-	-	-	-	-	-	-	-	-
060B20_3	0	AB811118	AB811287	F:TATGGTTGTTAAGCGTCAGAT R:TTTTTGTTATGGTAGTTATGATT	48	555	554	554	554/555 (99%)	550/554 (99%)	CAPS, ***Msp*I**	554	68, 486	NLL-12	17.5	24.7	2	2	2	[illegible]
060B20_5	0	AB811119	AB811288	F:CAATTCAACGTAACATAATATCG R:GTAAATATGCATCATCTGGAAC	51	503	501	501	501/503 (99%)	500/501 (99%)	-	-	-	-	-	-	-	-	-	-
060B20_5D	0	AB811120	AB811289	F:<u>AAAAGAATTATGAAATTACAAGTA</u> R:GTAAATATGCATCATCTGGAAC	50	292	292	292	290/292 (99%)	291/292 (99%)	dCAPS, ***Rsa*I**	292	24, 268	NLL-12	24.7	22.9	2	2	2	[illegible]
072M20_3	5	AB811121	AB811290	F:GTCATCTTCCATCACATTATCA R:GGAGAAAGTTTATGCAATCTGT	50	520	530	530	514/532 (96%)	530/530 (100%)	-	-	-	-	-	-	-	-	-	-
072O21_3	2	AB811122	AB811291	F:GATTCTGATTTCCAGTACGAAT R:CTTAGAGGCGTTCAGTCATAAT	50	410	409	409	409/410 (99%)	409/409 (100%)	-	-	-	-	-	-	-	-	-	-
072O21_3F2	2	AB811123	AB811292	F:GTTTGCCGACCAGCAGTAG R:ATAGGGAATGCGAGATGAG	50	742	740	740	738/742 (99%)	739/740 (99%)	CAPS, ***Mbo*II**	232, 508	740	NLL-16	12.1	25.0	2	1	1	[illegible]
072O21_3F2E	2	AB811124	AB811293	F:<u>TTCGTCCCTCCCATCTCAAACCTG</u> R:ATAGGGAATGCGAGATGAG	60	523	522	522	520/523 (99%)	522/523 (99%)	dCAPS, ***Bve*I**	29, 493	522	NLL-16	25.0	13.9	2	1	1	[illegible]
072O21_5	2	AB811125	AB811294	F:CGGAAATTAGATAACAAGTGTG R:TGTGAGATTTTTGCTTCTCTC	48	328	329	329	295/333 (88%)	329/329 (100%)	-	-	-	-	-	-	-	-	-	-
075D16_3	4	AB811126	AB811295	F:AGGCTGTGAGAAGAGAAACAT R:CTTTCCTAATCATTTCGTCTG	50	510	510	510	510/510 (100%)	509/510 (99%)	CAPS, ***Taq*I**	32, 478	510	NLL-03	20.8	22.9	1	1	1	[illegible]
075D16_5	4	AB811127	AB811296	F:AAAGCCCATTAGAAAATGAGTA R:AGAAACACCTCATTAATCAAAAA	46	367	365	365	365/367 (99%)	365/365 (100%)	-	-	-	-	-	-	-	-	-	-
080K03_3	12	AB811128	AB811297	F:TGACTGCATATACCCAACTATT R:ACCTAGTTGAATCTAACCGTGT	53	629	628	628	622/630 (99%)	625/628 (99%)	CAPS, ***Hpy*188I**	103, 110, 415	103, 525	NLL-12	22.9	17.5	2	2	2	[illegible]
080K03_5	12	AB811129	AB811298	F:AGCTTGTACCTTTAACTTTAACAC R:AGTGTTGTTTCGAAGAATTATG	53	501	499	499	499/501 (99%)	498/499 (99%)	-	-	-	-	-	-	-	-	-	-
080K03_5D	12	AB811130	AB811299	F:<u>AGTAAGCTTATCTTAACAACGAGC</u> R:AGTGTTGTTTCGAAGAATTATG	50	187	187	187	185/187(99%)	186/187(99%)	dCAPS, ***Alu*I**	7, 180	7, 17, 163	NLL-12	18.6	22.9	2	2	2	[illegible]
084P09_3	5	AB811131	AB811300	F:AAGCTTGAGAAACGTATTTTATTT R:AACAGTAAGAACAACGGTAACA	48	320	339	339	309/322(96%)	339/339(100%)	-	-	-	-	-	-	-	-	-	-
084P09_3F2	5	-	-	F:TGCTGTTACTGTTAACGCTGCT R:GCCTCCGTAAATCCCTAGT	69	484	sequence unclear	sequence unclear	-	-	-	-	-	-	-	-	-	-	-	-
084P09_5	5	AB811132	AB811301	F:GTCATCTTCCATCACATTATCA R:TTGTTGTAGAGCTATGTTCGAT	48	324	276	276	256/270 (94%)	276/276 (100%)	-	-	-	-	-	-	-	-	-	-
084P09_5F2	5	AB811133	AB811302	F:CATCGAACATAGCTCTACAACAA R:GATTGATAATAAGGTAGAGCCTCA	53	342	340	340	339/342 (99%)	340/340 (100%)	-	-	-	-	-	-	-	-	-	-
084P14_3	5	AB811134	AB811303	F:TAACTTGGATAAAATGCAAACTT R:TGAAAAATGCTCTCTTTTTACTT	50	404	402	402	382/404(95%)	402/402 (100%)	-	-	-	-	-	-	-	-	-	-
084P14_5	5	AB811135	AB811304	F:CAATGTCAGAATGAGAATCTTTT R:GCATAAATGCAATTTGATACTCT	48	234	236	236	234/236 (99%)	236/236 (100%)	-	-	-	-	-	-	-	-	-	-
084P14_5F2	5	AB811136	AB811305	F:CACTCATGAAAGAACCGAGGA R:TAGCGTAGGACAGTCCATCT	63	591	590	575	589/591 (99%)	573/590 (97%)	CAPS, ***Eco*130I**	171, 207, 212	212, 363	NLL-08	21.1	18.2	1	2	1	[illegible]
087H02_3	5	AB811137	AB811306	F:GTAACTGCCTGTTCTTCTACCT R:AACTTTTTGGTTCTTATGTGCT	49	425	425	425	422/425(99%)	425/425 (100%)	-	-	-	-	-	-	-	-	-	-
087H02_5	5	AB811138	AB811307	F:AAGCTTCCTCTTTGTTCAAGT R:CCACTTCCTAGGATTGGTATT	50	501	500	500	494/501 (98%)	500/500 (100%)	-	-	-	-	-	-	-	-	-	-
105I24_3	0	AB811139	AB811308	F:CAAACAAGGTTTAAATATCAAGG R:TCAATATTATCTAAGTATCCCATGAC	47	503	503	503	502/503 (99%)	498/503 (99%)	CAPS, ***Rsa*I**	204, 299	503	NLL-03	14.6	18.4	1	2	2	[illegible]
105I24_5	0	AB811140	AB811309	F:TTGATTTAAGATCCAAGAACTTT R:CATTTCAACATTTCATAATTGC	46	501	498	498	497/501 (99%)	498/498 (100%)	-	-	-	-	-	-	-	-	-	-
107M16_3	6	AB811141	AB811310	F:AATGGTATGGCTACCTGTTTT R:CAGACCTATGTTATGTTTTTACTG	50	501	501	501	501/501 (100%)	500/501 (99%)	CAPS, ***Tas*I**	28, 71, 89, 92, 108, 113	28, 89, 92, 108, 184	NLL-17	16.6	9.3	1	2	2	[illegible]
109D22_3	10	AB811142	AB811311	F:TTTTTCAAATCCTCATGTAGTTC R:ACATAGTTTTGCATTGTCTTGA	59	636	636	635	636/636 (100%)	630/636 (99%)	CAPS, ***Bsh*1236I**	335, 301	635	NLL-02	27.1	19.4	1	1	2	[illegible]
111L22_5	3	AB811143	AB811312	F:CCCTTGCTAACTTATTCTCTG R:GATGTCCATCCCAACAAG	53	517	515	515	515/517 (99%)	513/515 (99%)	CAPS, ***Bse*GI**	16, 499	16, 28, 471	NLL-17	24.4	10.9	2	1	2	[illegible]
112E01_3	9	AB811144	AB811313	F:CAGACTCAATTTTCAACCAATC R:AACAAGGTTTTTGTAGGTGAAC	50	517	533	533	483/536 (90%)	533/533 (100%)	-	-	-	-	-	-	-	-	-	-
112E01_5	9	-	-	F:GCTTCTCAAAATGGTTATTGAT R:ACAAACAAACAACCTTTCTCC	61	151	sequence unclear	sequence unclear	-	-	-	-	-	-	-	-	-	-	-	-
112F11_3	1	AB811146	AB811315	F:AAGCTTTTAGGCTTTTCTTTC R:ATACCCTGCTATATACTTGTTAAT	49	519	529	529	292/307 (95%)	529/529(100%)	-	-	-	-	-	-	-	-	-	-
112F11_5	1	AB811147	AB811316	F:AATCATCAAATCAAGAATCAAAG R:CTCCTTCCTCTTTCTACTTCCT	48	218	218	218	215/218(99%)	218/218 (100%)	-	-	-	-	-	-	-	-	-	-
112F11_5F2	1	AB811148	AB811317	F:AGAAAGAGGAAGGAGAAAGCAG R:CAGCAATCTATAAACTGATTTTGTCT	53	671	670	670	670/671 (99%)	670/670 (100%)	-	-	-	-	-	-	-	-	-	-
112N18_3	8	AB811149	AB811318	F:TGGAACTTGATTTTTAAGTCCT R:CTTATCTACATTACCGCAACAT	51	502	502	502	498/505 (98%)	501/502 (99%)	-	-	-	-	-	-	-	-	-	-
112N18_3D	8	AB811150	AB811319	F:TGGAACTTGATTTTTAAGTCCT R:<u>ATTACCGCAACATCAAATTTCCGTA</u>	50	493	493	493	488/496(98%)	492/493(99%)	dCAPS, ***Rsa*I**	493	23, 470	NLL-14	24.7	8.4	1	1	1	[illegible]
112N18_5	8	AB811151	AB811320	F:GTTTGACATCGGTAATGTTAAT R:AGCAAGACAGATGCCATATT	49	558	557	557	557/558 (99%)	557/557 (100%)	-	-	-	-	-	-	-	-	-	-
115C21_3	1	AB811152	AB811321	F:GCTTATCTGCTTTGTGCATGAAT R:ATTTTCTTCACTCAACCCCTCT	60	533	531	531	530/534 (99%)	531/531 (100%)	-	-	-	-	-	-	-	-	-	-
115C21_5	1	AB811153	AB811322	F:GTTAAGCTCATAATCACCACAGC R:TACTACCAAGAGCATTCGCTTAC	57	255	253	253	246/256 (97%)	250/253 (99%)	CAPS, ***Bst*UI**	253	85, 168	NLL-14	18.9	12.7	1	1	1	[illegible]
115G22_3	2	AB811154	AB811323	F:CCCGGAAATTAGATAACAAGTG R:GCACACGGTAGAGACAAAGTCA	64	713	713	713	713/713(100%)	713/713(100%)	-	-	-	-	-	-	-	-	-	-
115G22_3F2	2	AB811155	B811324	F:GACACCATGAACCACACACAA R:TTTGCACTTTCAAGTTTTACGA	52	626	626	626	626/626(100%)	625/626(99%)	-	-	-	-	-	-	-	-	-	-
115G22_5	2	AB811156	AB811325	F:CTTCCTTACATTTGTCCATTTCA R:TTATGACGAAACCTACGCTCCT	65	564	562	562	557/564(99%)	562/562(100%)	-	-	-	-	-	-	-	-	-	-
115G22_5F2	2	AB811157	AB811326	F:GATACCCTCCTCCTGGTTGTAG R:AAGCCGATGAGGGAATATTTTT	52,5	605	606	606	595/607(98%)	601/606(99%)	-	-	-	-	-	-	-	-	-	-
115G22_5F3	2	AB811158	AB811327	F:GGATAGACTCTTCAACTTGCATT R:ACGGGGGAGAATTTGAGAAC	49	453	451	451	444/453(98%)	451/451(100%)	-	-	-	-	-	-	-	-	-	-
115G22_5F4	2	AB811159	AB811328	F:TTTTTCCTCTCTACAACTCCATTTT R:AGCCAACTTTGCGATAAAGGTA	60	698	692	692	689/698(99%)	692/692(100%)	-	-	-	-	-	-	-	-	-	-
119M13_3	7	AB811160	AB811329	F:GGACTTGTTCCTCTAAGATTGG R:CACGAGTGCTATTAACCTCTCA	57	621	616	616	615/621 (99%)	615/616 (99%)	-	-	-	-	-	-	-	-	-	-
119M13_3D	7	AB811161	AB811330	<u>F:GCGTTTTGGATGTTTTTAAAAGCATGCCT</u> R:CACGAGTGCTATTAACCTCTCA	57	410	410	410	408/410(99%)	409/410(99%)	dCAPS, ***Bfa*I**	44, 155, 211	28, 44, 155, 183	NLL-04	17.9	11.8	1	1	2	[illegible]
119M13_5	7	AB811162	AB811331	F:GCTTGATCCTTTTTATGGTTG R:CCAGAGGGAAGAATTGTACATC	60	503	502	502	499/503 (99%)	501/502 (99%)	-	-	-	-	-	-	-	-	-	-
119M13_5D	7	AB811163	AB811332	<u>F:TAGAATTTTGATAAATTATATTTTTTG</u> R:CCAGAGGGAAGAATTGTACATC	48	345	345	345	341/345(99%)	344/345(99%)	dCAPS, ***Cvi*RI**	27, 44, 55, 71, 91, 128	27, 44, 55, 91, 128	-	-	-	-	-	-	-
120E23_5	0	-	-	F:AGCTTGGGCGAGAGTAGT R:GGGATTATTTGGGCTTGA	56	616	616	616	not sequenced	not sequenced	CAPS, ***Tru*1I**	not sequenced	not sequenced	NLL-02	17.1	16.7	0	1	2	[illegible]
123A20_3	14	AB811164	AB811333	F:GCTTTACCTCAGTCCTATCTTT R:ACCACAAAGTGTTAGAAAATGA	53	506	503	503	501/506 (99%)	503/503 (100%)	-	-	-	-	-	-	-	-	-	-
123A20_3F2	14	AB811165	AB811334	F:ATATATGGAATAATCAACACTGTCC R:CATAGGCATATATCCGTTATGCT	56	299	299	299	299/299(100%)	299/299(100%)	-	-	-	-	-	-	-	-	-	-
123A20_5	14	AB811166	AB811335	F:TTTACCTTCACCCACTTCTAAT R:TTGATTAATGGTAGTTAGAGTTTCT	56	625	624	624	601/625 (96%)	624/624 (100%)	-	-	-	-	-	-	-	-	-	-
123A20_5F2	14	AB811167	AB811336	F:TGATTAACATATTCCCGTTTTCG R:AACATTGAACTAACGTAATGAG	57	244	244	244	244/244(100%)	242/244(99%)	-	-	-	-	-	-	-	-	-	-
123A20_5F3	14	AB811168	AB811337	F:TCAGTAACCCATTATGTGAGTGAG R:CAGACTGAGTTACAAAATTGACC	47	573	568	568	557/573(97%)	566/568(99%)	-	-	-	-	-	-	-	-	-	-
128I22_3	0	AB811169	AB811338	F:TGAAAATTCTCGTTTACTATGG R:TTGCAAAGTATGTTGGATATAA	48	551	548	548	548/551 (99%)	548/548 (100%)	-	-	-	-	-	-	-	-	-	-
128I22_3F2	0	AB811170	AB811339	F:GCAAGAATCACCATTTGCCTCA R:CGGACCAGTCGAGTCAATCAA	60	607	607	607	607/607 (100%)	607/607 (100%)	-	-	-	-	-	-	-	-	-	-
128I22_3F3	0	AB811171	AB811340	F:TCACAGTTGAATCGGTCGAA R:TGGTTGTACTGTCAGAATCCAAA	60	595	595	595	593/595 (99%)	595/595 (100%)	-	-	-	-	-	-	-	-	-	-
128I22_5	0	-	-	F:AAATGAAGGATATGGGTGAGAC R:ATTTCCTTGAGTCCATACATCG	57	470	sequence unclear	sequence unclear	-	-	-	-	-	-	-	-	-	-	-	-
128I22_5F2	0	-	-	F:ATATCAAAGTGATCCTGAAATGG R:GACACCTTATGTTTTCGTACTTATTCT	51	468	sequence unclear	sequence unclear	-	-	-	-	-	-	-	-	-	-	-	-
128I22_5F3	0	AB811172	AB811341	F:ACATGTATGGATGTTTTCTTATCATT R:CCCATTGGCATATACTCAACAC	55	438	438	438	437/438 (99%)	436/438 (99%)	CAPS, ***Nla*III**	2, 38, 83, 101, 214	2, 38, 184, 214							
131C21_3	0	AB811173	AB811342	F:AGCTTTTTCGTTTTGTCATT R:CAAGACTGACATTATTTTGACC	52	467	451	458	450/467 (96%)	446/458 (97%)	CAPS, ***Nde*I**	151, 300	458	NLL-01	18.7	23.8	1	2	1	[illegible]
131C21_5	0	-	AB811343	F:AAGCTTTGATGAGAAACATGA R:CTTAAGAAAGGTACAAGGGATT	56	451	-	450	-	449/452 (99%)	AS-PCR	-	450	NLL-01	14.8	18.7	1	2	1	[illegible]
136B16_3	8	AB811174	AB811344	F:AAGCTTTCTTAATACCCAAGAC R:CAGAAAAATGTAATGAAAGTGG	58	534	531	533	458/545 (84%)	504/534 (94%)	CAPS, ***Taq*I**	78, 163, 290	19, 52, 60, 78, 161, 163	-	-	-	-	-	-	-
136B16_5	8	AB811175	AB811345	F:AAGCTTGTTTCGAATTGTCTAT R:GCCACAATTTCAAGCTGTTA	49	452	449	449	447/453 (98%)	448/449 (99%)	CAPS, ***Mva*I**	157, 292	449	NLL-14	17.6	24.7	1	1	1	[illegible]
138M02_3	7	AB811176	AB811346	F:GCTTTGATAGAACCTTGTCTTCA R:CTGCCCTAATGCTCATCCTAT	60	163	164	164	162/166 (98%)	162/164 (99%)	-	-	-	-	-	-	-	-	-	-
138M02_3D	7	AB811177	AB811347	<u>F:GATGATATGATGAGGATGAGGAGGAGGC</u> R:CTGCCCTAATGCTCATCCTAT	-	-	no PCR product	no PCR product	-	-	-	-	-	-	-	-	-	-	-	-
138M02_5	7	-	-	F:GAATTTACATTTTTGCCCTGTC R:GGCACAACCACAAAAAGTTTC	63	329	sequence unclear	sequence unclear	-	-	-	-	-	-	-	-	-	-	-	-
138N02_3	0	AB811178	AB811348	F:GATGTTATGGAACAACGTCTCA R:ACCAAACATGATCAAGATCCAC	57	559	561	561	549/562 (98%)	561/561 (100%)	-	-	-	-	-	-	-	-	-	-
138N02_3F2	0	AB811179	-	F:TCAATTTTCTTACAACTCGCTGA R:AGAGAACAACTGCACAAATGAA	65	413	413	-	413/413(100%)	-	AS-PCR	-	413	NLL-14	8.4	10.4	1	2	1	[illegible]
138N02_5	0	AB811180	AB811349	F:TGAGATCCTCTTGTGACATAGC R:ACTCATAAAATCCTCTCCCAAG	55	255	253	253	250/255 (98%)	253/253 (100%)	-	-	-	-	-	-	-	-	-	-
141C03_5	3	AB811181	AB811350	F:TTAGGCTTCCCCTTGCTA R:CCTTACACCACTAGACCAAC	56	451	448	448	446/451 (98%)	447/448 (99%)	-	-	-	-	-	-	-	-	-	-
141C03_5D	3	AB811182	AB811351	F:<u>TTTTTCTACTCTCTATACATAATAGC</u> R:CCTTACACCACTAGACCAAC	41	274	274	274	270/274(99%)	273/274(99%)	dCAPS, ***Alu*I**	25, 249	274	NLL-17	18.6	21.4	2	1	2	[illegible]
